# Serial dependencies between locomotion and visual space

**DOI:** 10.1038/s41598-023-30265-z

**Published:** 2023-02-27

**Authors:** Michael Wiesing, Eckart Zimmermann

**Affiliations:** Institute for Experimental Psychology, Heinrich Heine University Duesseldorf, Düsseldorf, Germany

**Keywords:** Perception, Motor control, Sensorimotor processing, Sensory processing, Visual system, Human behaviour

## Abstract

How do we know the spatial distance of objects around us? Only by physical interaction within an environment can we measure true physical distances. Here, we investigated the possibility that travel distances, measured during walking, could be used to calibrate visual spatial perception. The sensorimotor contingencies that arise during walking were carefully altered using virtual reality and motion tracking. Participants were asked to walk to a briefly highlighted location. During walking, we systematically changed the optic flow, i.e., the ratio between the visual and physical motion speed. Although participants remained unaware of this manipulation, they walked a shorter or longer distance as a function of the optic flow speed. Following walking, participants were required to estimate the perceived distance of visual objects. We found that visual estimates were serially dependent on the experience of the manipulated flow in the previous trial. Additional experiments confirmed that to affect visual perception, both visual and physical motion are required. We conclude that the brain constantly uses movements to measure space for both, actions, and perception.

## Introduction

How do we estimate the distance of objects in front of us? Already in 1709, Berkeley emphasized that body motion implicitly measures external space, thus providing signals to build up visual space^1^. This hypothesis requires that motor-related signals about body movement to be transferred to areas that generate visual perception.

Locomotion produces predictable sensory consequences, such as optic flow. During walking, predictions are compared to the actual sensory input to guide behavior^2^. Idiothetic signals, i.e., information resulting from self-motion, such as proprioceptive, visual cues or efference copies of motor commands, modulate the activity in the primary visual cortex (V1)^3^ as well as the self-position within the environment^4,5^. Active navigation through an environment has also been found to modulate the amplitude of responses along the visual pathway^4^. Some evidence suggests that activity in the primary visual cortex of behaving mice is mainly modulated by non-visual input. Using two-photon imaging, Keller et al.^6^ found that when comparing the calcium activity in the visual cortex during self-generated optic flow and the playback of previously recorded optic flow, the majority of the activity was related to motor activity rather than visual stimulation. They also found that when they inserted trials with short periods of no optic flow, the mismatch between visual and physical motion was a better predictor of activity in the primary visual cortex than visual input alone. These findings clearly show that motor-related signals modulate activity in primary visual cortex and may provide the physiological basis for visual space recalibration.

In rodents it has also been demonstrated that activity in V1 is correlated with activity in the hippocampus, even in the absence of visual feedback^7^. V1 activity is modulated by physical distance traveled, possibly carrying top-down prediction signals about the spatial scene^8^. Hence, the traveled distance could be compared to the initially visually predicted distance, with the resulting error used to recalibrate visual space.

Here we examined if prediction errors caused by a mismatch between the visually estimated egocentric distance of a target and the actual traveled distance are used to recalibrate visually perceived space. We used participants' sensitivity to changes in optic flow gain to manipulate travel distances. A mismatch between visual and physical motion is known to bias subsequent distance estimates in blind walking^9,10^. Rieser et al.^10^, for example, manipulated the optic flow by having participants walk on a treadmill located on a trailer pulled by a tractor. This enabled the authors to separate the visual motion speed (i.e., tractor speed) from the physical motion speed (i.e., speed of the treadmill). Participants overshot the target location in subsequent blind walking trials after adaptation to visual motion being slower than physical motion, whereas the opposite was found after adaptation to faster visual motion than physical motion.

Some behavioral evidence in human participants indicating recalibration of perceived space because of walking through an environment comes from virtual reality research (VR). Distances in virtual environments (VEs) are frequently underestimated, as evidenced by numerous studies^11,12^. While the exact causes for distance compression in VEs are not fully understood, a brief period of physically walking through a VE with visual feedback has been found to reduce distance compression in subsequent blind walking trials^13^. Interestingly, when stationary participants viewed simulated walking, these effects were not observed, indicating that physically walking through the VE is required. Walking through a VE with visual feedback has also been found to improve verbal distances estimates^9^ (but see Kelly et al.^14^ and Kunz et al.^15^) or judgements of object size^16,17^. In these studies, effects of visuomotor feedback on visually perceived distance or size have been observed after many adaptation trials (typically about 15–20 trials). However, results by Kelly et al.^18^ indicate that recalibration of perceived distances occurs already after a few trials. Theoretically, if the information gained from walking to a visual target is used to recalibrate perceived space, recalibration effects should occur already after a single trial.

Recently, we showed that post-saccadic errors calibrate visual localization^19^. Following artificially induced post-saccadic errors, subsequent saccades and visual localization were attracted by the sensed target position. This recalibration process operates on a trial-by-trial—basis revealing the existence of serial dependencies from action on visual perception that were previously observed in visual perception^20,21^, motion extrapolation^22,23^ or for head movements^24^.

We asked participants to walk to a briefly highlighted location 2.50 m ahead of them and to stop when they thought to stand at the correct location. Unbeknownst to the participants, the ratio of visual and physical motion speed was randomly changed across trials with factors between 0.8 and 1.2 in 0.1 steps by using a so-called two dimensional translation gain^25,26^. As a result, the visual distance to the target remained identical across trials, while the physical travel distance to reach the target location, varied on a trial-by-trial basis.

We expected the perturbed visual motion speed to bias the walking distance estimates based on previous research. We hypothesized that exposure to visual motion being slower than physical motion would result in longer travel distances than increased visual motion. We hypothesized that these recalibration effects would transfer to visually estimated distances in a serially dependent manner. We expected to see visual distance estimates influenced by the previous walking trial's travel distance. Hence, after trials with short travel distances, we expected participants to estimate egocentric distance to be shorter in visual judgement trials and relatively longer in walking trials.

## Results

### Experiment 1

**Figure 1:**
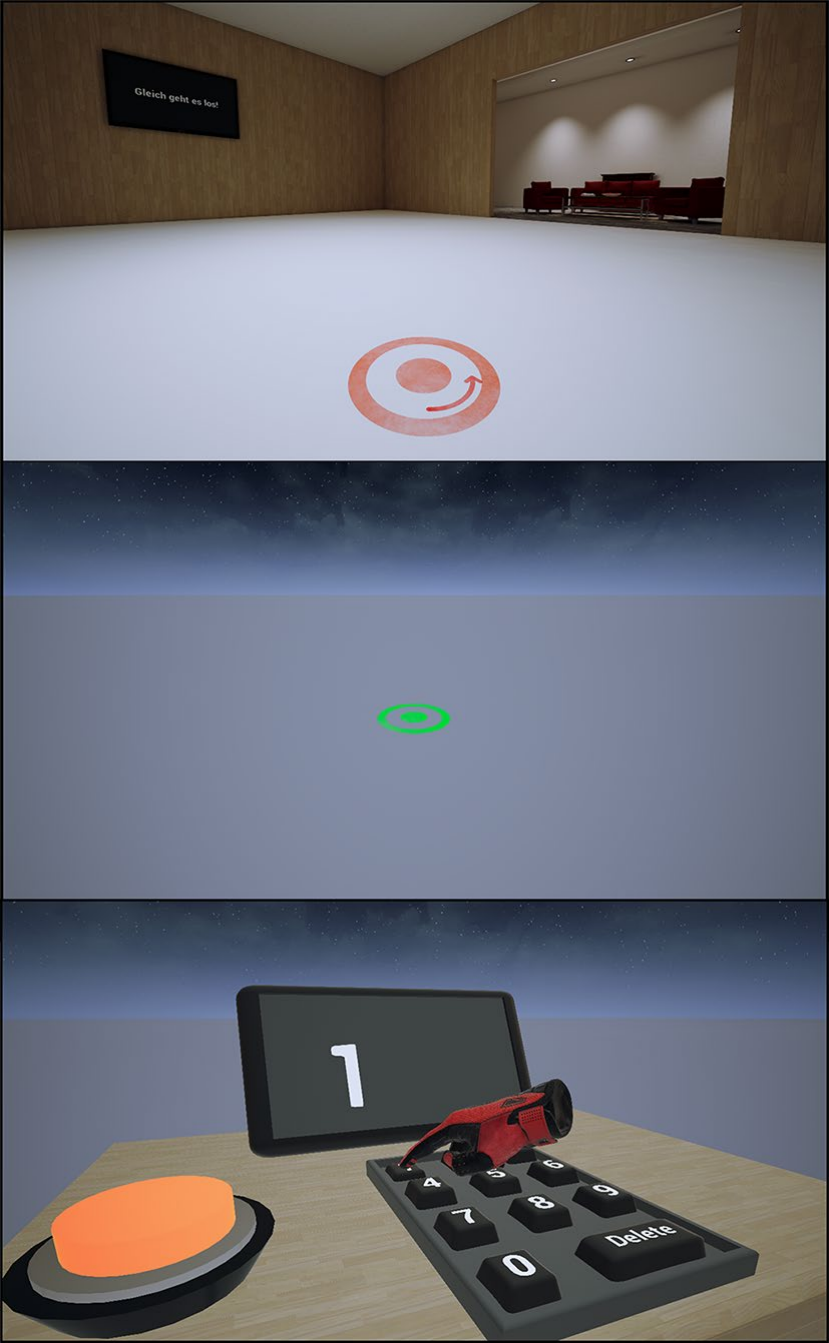
Screenshots of the virtual room environment (upper panel) and the ground plane environment (middle panel) that were custom created in Unreal Engine 4.25 for this research project and used in the experiments. Visual distance estimates were entered by the participants via a virtual numeric keyboard (lower panel).

**Figure 2:**
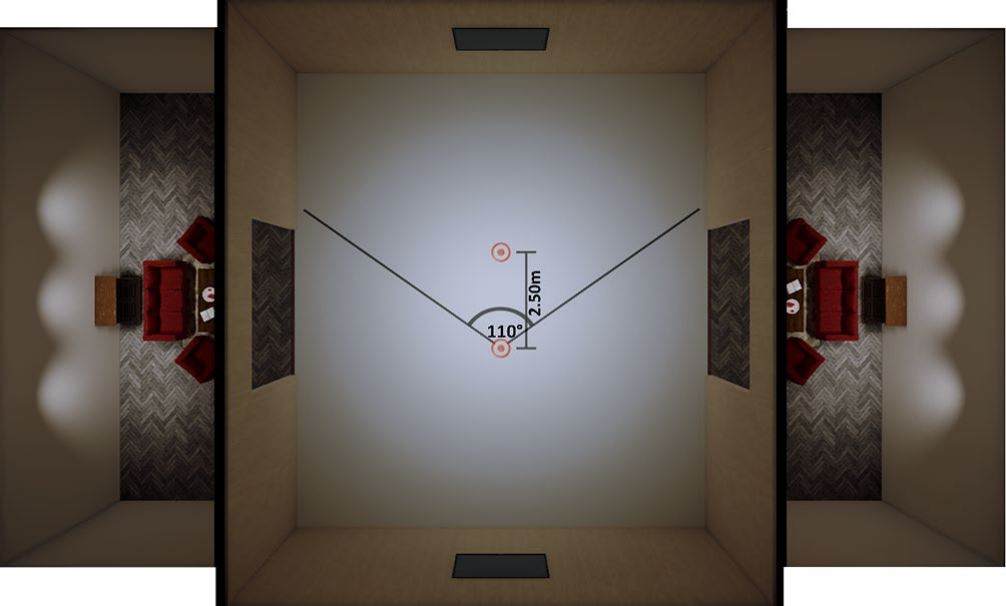
Top view on the room environment (created in Unreal Engine 4.25). The door to the living rooms on both sides had a size that ensured that they were outside of the field of view, when participants were looking forwards, hence preventing them from causing additional motion cues such as parallax (created in UE4).

Participants wore an HTC Vive Pro Eye head-mounted display (HMD) and performed a walking distance estimation task followed by a visual distance estimation task. To measure sensorimotor serial dependencies between action and perception we implemented a trial structure in which walking and visual judgement trials alternated. Walking trials were conducted in a realistic, rendered indoor environment (Fig. 1, upper panel and Fig. 2). Participants saw a flat red circle (diameter: 50 cm) flashing up for 200 ms, 2.50 m in front of them on the ground. Their task was to walk to the location where they have seen the target and indicate by button press when they thought to be at the correct location. After each walking trial (trial n_W_ − 1) participants turned around and faced the previously walked path for the visual judgement trial. Before starting the trial, the indoor environment disappeared, and participants found themselves in a monotonous ground plane environment (Fig. 1, middle panel). The environment was designed to minimize available spatial landmarks, which otherwise might interfere with the visual distance estimates. Participants were asked to estimate the egocentric distance between themselves and a target stimulus, which was presented as a flat green circle (diameter: 50 cm) at a distance between 0.80 and 1.80 m (0.2 m steps) for 33 ms on the ground. Apart from the color, the target was identical to the red target from the walking task. After the target disappeared, a table with a small display and a virtual keyboard, consisting of numeric buttons for all digits from 0 to 9, a delete and a confirm button, appeared behind the participant. Participants were asked to enter their distance estimate into the numeric keyboard in centimeters using their right index-finger (Fig. 1, lower panel).

Previous research has repeatedly shown that participants underestimate egocentric distances in virtual environments^12,14,27^. Hence, in a first step, we assessed if distance estimates of walking and visual judgement trials were affected by distance compression. We calculated the mean judged-to-distance ratio i.e., the estimated distance divided by the actual distance, for all trials with a gain = 1.0, i.e., no conflict between visual and physical motion, separately for both walking distances and visual distance judgements for each participant. On average, we observed judged-to-distance ratio of 1.02 (SD = 0.05) for walking distance estimates. A one sample t-test to examine if the judged-to-distance ratios differ from 1, did not provide evidence for a difference between the traveled distance estimates and the actual travel distance (t(19) = − 1.62, p = 0.12, *d* = 0.36). On the other hand, egocentric distances were underestimated in visual judgement trials (M = 0.87, SD = 0.13), indicating distance compression. This was confirmed by a significant t-test (t(19) =  − 4.13, p < 0.001, *d* = 0.88), in line with previous research^14^.

**Figure 3:**
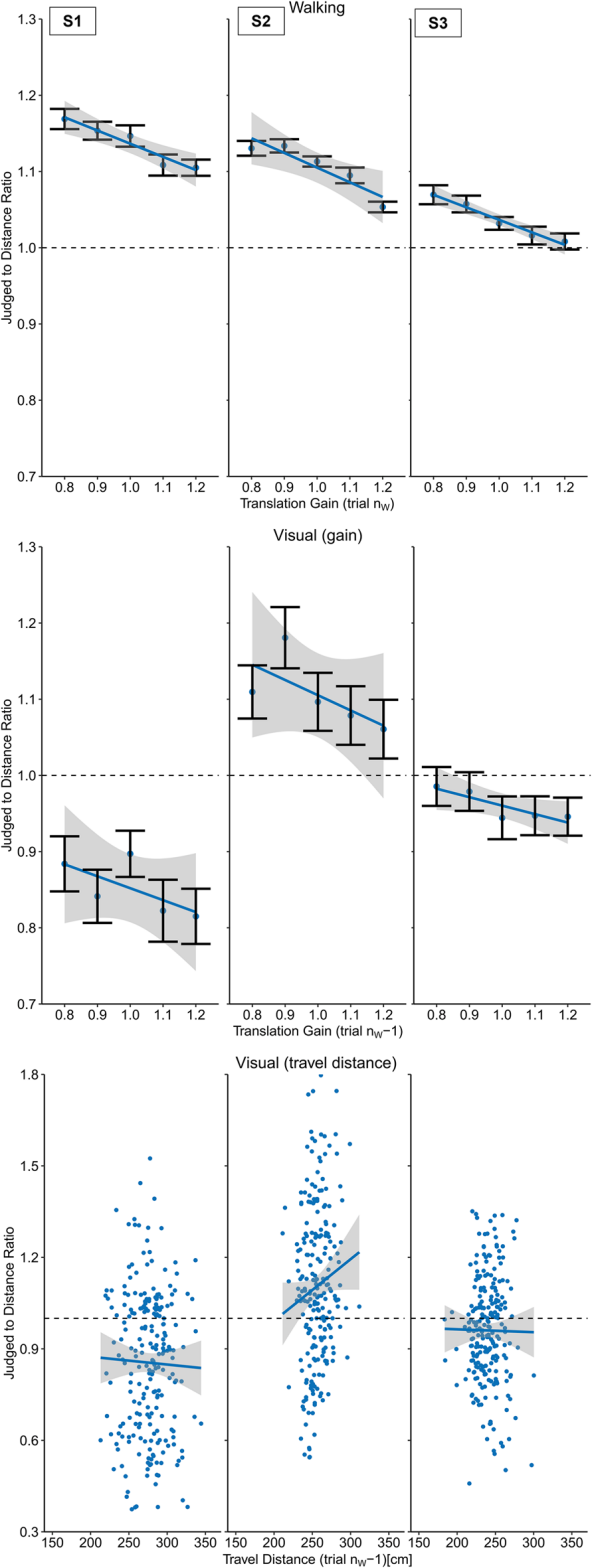
Results of Experiment 1 (physical walking with optic flow) for three example participants. Upper panel: Regression between the translation gain in trial n_W_ and judge to distance ratio for walking distance estimations in trial n_W_. Middle panel: Serial dependencies quantified by the regression between the translation gain in in trial n_w_ − 1 and visual distance judgements. Lower panel: Serial dependencies quantified by the regression between travel distance in trial n_w_ − 1 and visual distance judgements. Error bars represent the SEM and the grey areas represent the 95% confidence interval.

In the next step we analyzed the effect of the translation gain on walking distance estimates. We found a clear negative slope of the regression between the translation gain and the travel distance (Slope: M = − 0.15, SD = 0.064) (Figs. 3 and 6). As expected, we observed shorter travel distances when visual motion was faster than physical motion and longer walking distances when visual motion was slower than physical motion (t(19) =  − 10.78, p < 0.001, *d* = 2.41).

After each walking trial (trial n_W_ − 1) participants had to visually localize an object presented between 0.80 and 1.80 m in front of them (trial n_V_). We found a robust serial dependence between the translation gain in trial n_W_ − 1 and subsequent visual distance estimates in trial n_V_ (Slope: M = − 0.05, SD = 0.002), (t(19) =  − 2.30, p < 0.05, *d* = 0.51), (Figs. 3 and 7), showing that visual distance estimates were modulated by the mismatch between visual and physical motion. These results indicate that visual judgements were biased by idiothetic information from locomotion. In order to examine the role of the previously traveled distance we analyzed visual judgements as a function of the travel distance in the previous walking trial n_W_ − 1 (Slope: M = − 7.98 × 10^–5^, SD = 0.10), which did not result in significant effect (t(19) = 0.21, p = 0.83, *d* = 0.05) (Figs. 3 and 9), providing no evidence that the travel distance influenced visual distance estimates.

In a next step, we tested the effect of the *target error* n_W_ − 1, i.e., the distance between participant and shifted target location at the end of the walking trial on the following visual distance judgement. Here a negative target error refers to trials in which participants undershot the perturbed target location and positive errors refer to trial where participants walked too far. First, as a sanity check the target error was analyzed as a function of the translation gain, which revealed a strong positive slope (Slope: M = 205.93, SD = 16.27) (t(19) = 56.59, p < 0.001, *d* = 12.65), indicating that a translation gain above 1.0 resulted in an overshooting of the target, while a translation gain lower than 1 had the opposite effect (Figs. S1, S11). Afterwards, we analyzed visual distance judgements in trial n_v_ as a function of the target error in trial n_W_ − 1. The analysis resulted in a negative slope (Slope: M = − 1.97 × 10^–4^, SD = 4.56 × 10^–4^) which was not significantly different from zero (t(19) =  − 1.93, p = 0.07, *d* = 0.43) (Figs. S1 and S12).

Previous research results indicate that optic flow affects the preferred walking speed of participants^28,29^, suggesting that increasing the optic flow speed results in reduced walking speed and vice versa. Hence, here we included post-hoc analyses of the maximum walking speed.

In the first step, a trajectory for each walking trial was calculated based on the location measurements of the Vive Tracker. The data were filtered for noise using a 3th order Savitzky-Golay smooting filter and a frame length of 31 samples (sampling rate 90 Hz). Only the positional data along the x- and z-axis was extracted and filtered. Afterwards the maximum speed was extracted for each trial.

We observed a negative slope of the regression of the translation gain on the maximum walking speed (t(19) =  − 5.889, p < 0.001, *d* = 1.32), indicating that participants reduce their preferred walking speed with increasing optic flow, thus confirming previous research (Figs. S5, S9).

However, we did not observe serial dependencies of the maximum walking speed in trial n_W_ − 1 on the visual distance estimates in trial n_v_ (t(19) = 1.94, p = 0.07, *d* = 0.42) (Figs. S5, S10).

Taken together, the results of Experiment 1 clearly demonstrate that exposure to optic flow, that is slower or faster than expected, directly influences walking distances estimates. As expected, participants traveled farther distances when visual motion was slower than physical motion and vice versa. We found evidence for serial dependencies between the recalibration of walking task and visual judgements. Following walking with reduced visual motion, participants visually estimated the target to be farther away and to be closer after walking with increased physical motion. However, we did not find evidence for a direct modulation of the travel distance on visual space.

### Experiment 2

Generally, two sorts of signals were available in Experiment 1 to measure external space which could be used to recalibrate visual space: the optic flow and processes related to the walking itself, such as the number of steps or duration of walking. In Experiment 2, we aimed to examine the role of the optic flow on effects on visual distance judgements observed in Experiment 1. While the procedure was essentially identical to Experiment 1, participants remained stationary during the walking task, but controlled their movement virtually using the thumb stick of the left controller. Previous research has shown that participants are able to estimate travel distances from optic flow in the absence of other motion cues^30,31^. Here we used this method to find out whether walking distances derived from visually perceived self-motion alone, in the absence of body-based motion cues, similarly recalibrate visual perceived distances. Again, the visual motion speed was changed randomly on a trial-by-trial basis, to mimic the translation gain of Experiment 1. In Experiment 2, a gain of 1.0 corresponds to a maximum movement speed of 1.4 m/s. The locomotion speed was controlled by the amount the thumb stick was pressed forward. Prior to each session, participants completed 14 training trials at a gain of 1.0 to get familiar with the medium movement speed.

We observed a significant overshooting of the target location across all trials with a gain = 1.0 in Experiment 2 (M = 1.07, SD = 0.06) (t(19) = 5.33, p < 0.001, *d* = 1.20). For the visual judgement trials, we did not find evidence for distance compression (M = 0.95, SD = 0.46), (t(19) = − 0.48, p = 0.63, *d* = 0.11).

**Figure 4:**
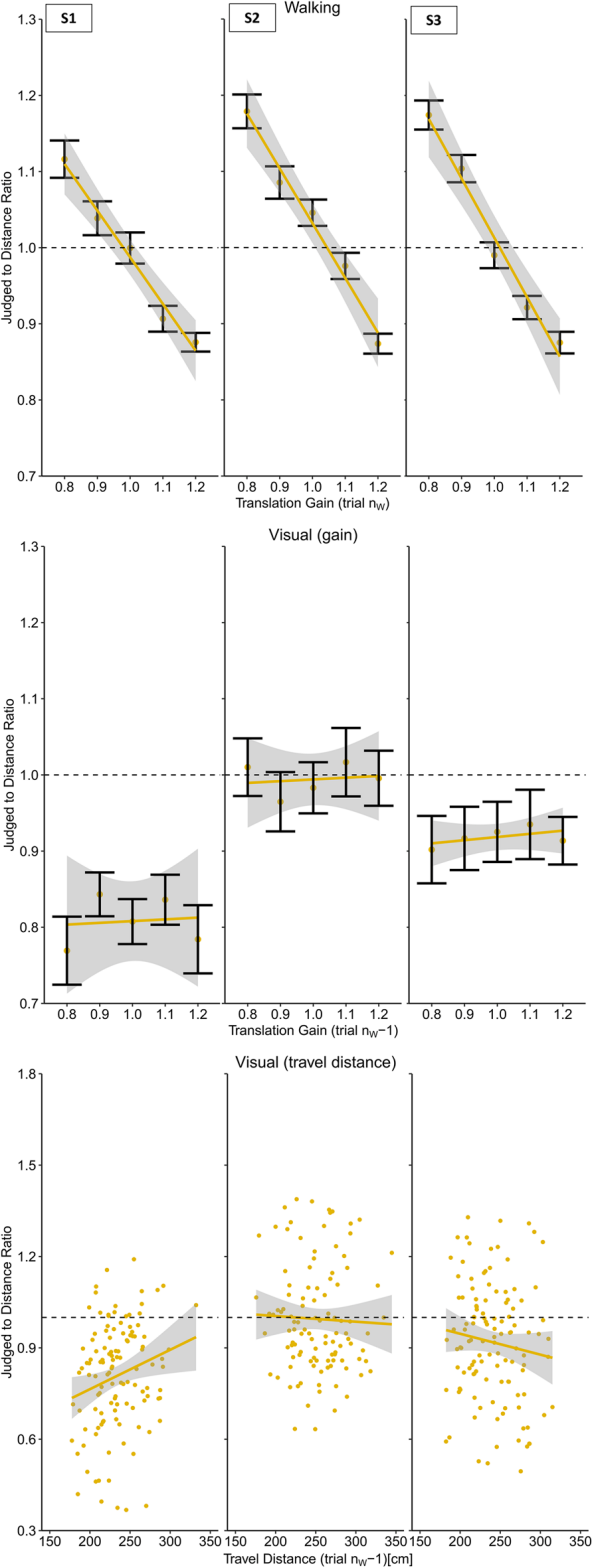
Results of Experiment 2 (artificial walking with optic flow) for three example participants. Upper panel: Regression between the translation gain in trial n_W_ and judge to distance ratio for walking distance estimations in trial n_W_. Middle panel: Serial dependencies quantified by the regression between the translation gain in in trial n_w_ − 1 and visual distance judgements. Lower panel: Serial dependencies quantified by the regression between travel distance in trial n_w_ − 1 and visual distance judgements. Error bars represent the SEM and the grey areas represent the 95% confidence interval.

We found a clear modulation of the visual motion speed on the travel distances (Slope: M = − 0.82, SD = 0.23), (t(19) =  − 16.273, p < 0.001, *d* = 3.64) (Figs. 4 and 6). Like Experiment 1, increased visual motion speed resulted in shorter travel distances and vice versa. In fact, the effect was drastically increased when compared to Experiment 1, which is most likely explained by the fact that body-based motion cues were absent and only visual motion cues were available to estimate the travel distance. Conflicting cues that could tell the actual travel distance, such as vestibular, proprioceptive^32^ were not available in Experiment 2. The strong and systematic distance misestimation indicates that subjects had a clear idea about how much optic flow to perceive to reach the target.

If the effects on visual judgements in Experiment 1 were driven by visual self-motion cues, we expected to also see stronger modulations of visual judgements. However, we did not find evidence for transfer to visually perceived distances, neither when analyzed as a function of the gain (Slope: M = 0.01, SD = 0.14), (t(19) = 0.38, p = 0.71, *d* = 0.08) (Figs. 4 and 7) nor as a function of the visually traveled distance in trial n_W_ − 1 (Slope: M = 4.26 × 10^–4^, SD = 0.001), (t(19) = 1.89, p = 0.07, *d* = 0.42) (Figs. 4 and 9), suggesting that visual self-motion cues alone are not sufficient to recalibrate visual space.

Again we observed a strong positive regression of the target error as a function of the translation gain (Slope: M = 64.83, SD = 45.77) which was significant different from zero (t(19) = 6.33, p < 0.001, *d* = 1.42) (Figs. S2 and S11). However, like in Experiment 1, we did not observe serial dependencies of the target error in trial n_W_ − 1 on visual distance judgements in trial n_v_ (Slope: M = 4.02 × 10^–4^, SD = 1.12 × 10^–2^) (t(19) = 1.80, p = 0.09, *d* = 0.40) (Figs. S2, S12).

We observed a negative slope for the regression of the translation gain on the maximum locomotion speed (t(19) =  − 2.72, p < 0.05, *d* = 0.61) (Figs. S6, S9), indicating that participants reduced the locomotion speed with increasing optic flow. However, again we did not observe an effect of the maximum walking speed on visual judgements (t(19) =  − 1.50, p = 0.15, *d* = 0.33) (Figs. S6, S10).

### Experiment 3

To isolate the physical walking, in Experiment 3, we eliminated the optic flow swapping the environments in which the tasks were completed. Hence, participants walked through the ground plane environment (Fig. 1, middle panel), while visual judgement were performed in the indoor environment (Fig. 1, upper panel). Hence, due to the absence of optic flow in the ground plane environment, participants did not receive visual self-motion cues. Instead, we provided terminal feedback about the performance after each walking trial. Before each walking trial, participants walked within the room environment to the start location, before the environment changed. After walking, the room environment reappeared together with the walking target. However, the environment and target stimulus were displaced relative to the initial location on a trial-by-trial basis by the same factors as used for the translation gain in Experiment 1. We expected that the perturbed error feedback of a walking trial n_W_ − 1 would bias the travel distance in the following walking trial n_W_.

**Figure 5:**
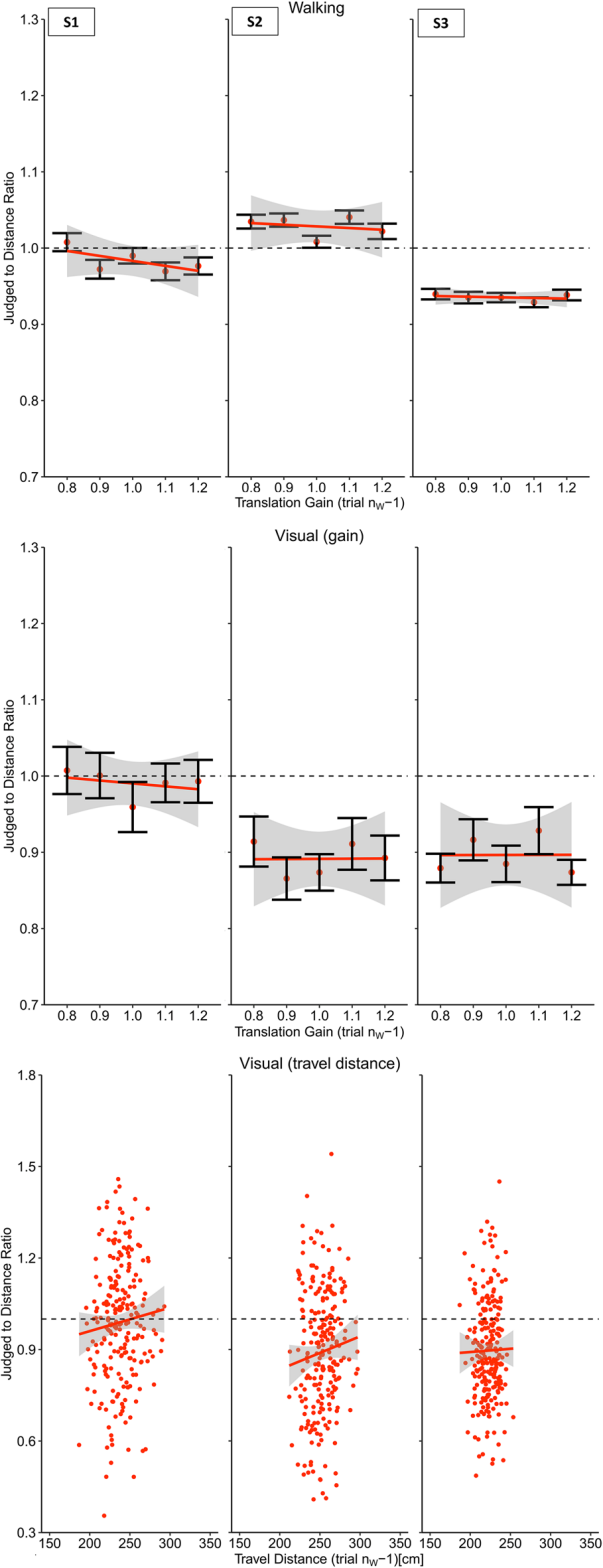
Results of Experiment 3 (physical walking without optic flow) for three example participants. Upper panel: Regression between the translation gain in trial n_W_ and judge to distance ratio for walking distance estimations in trial n_W_ − 1. Middle panel: Serial dependencies quantified by the regression between the translation gain in in trial n_w_ − 1 and visual distance judgements. Lower panel: Serial dependencies quantified by the regression between travel distance in trial n_w_ − 1 and visual distance judgements. Error bars represent the SEM and the grey areas represent the 95% confidence interval.

**Figure 6:**
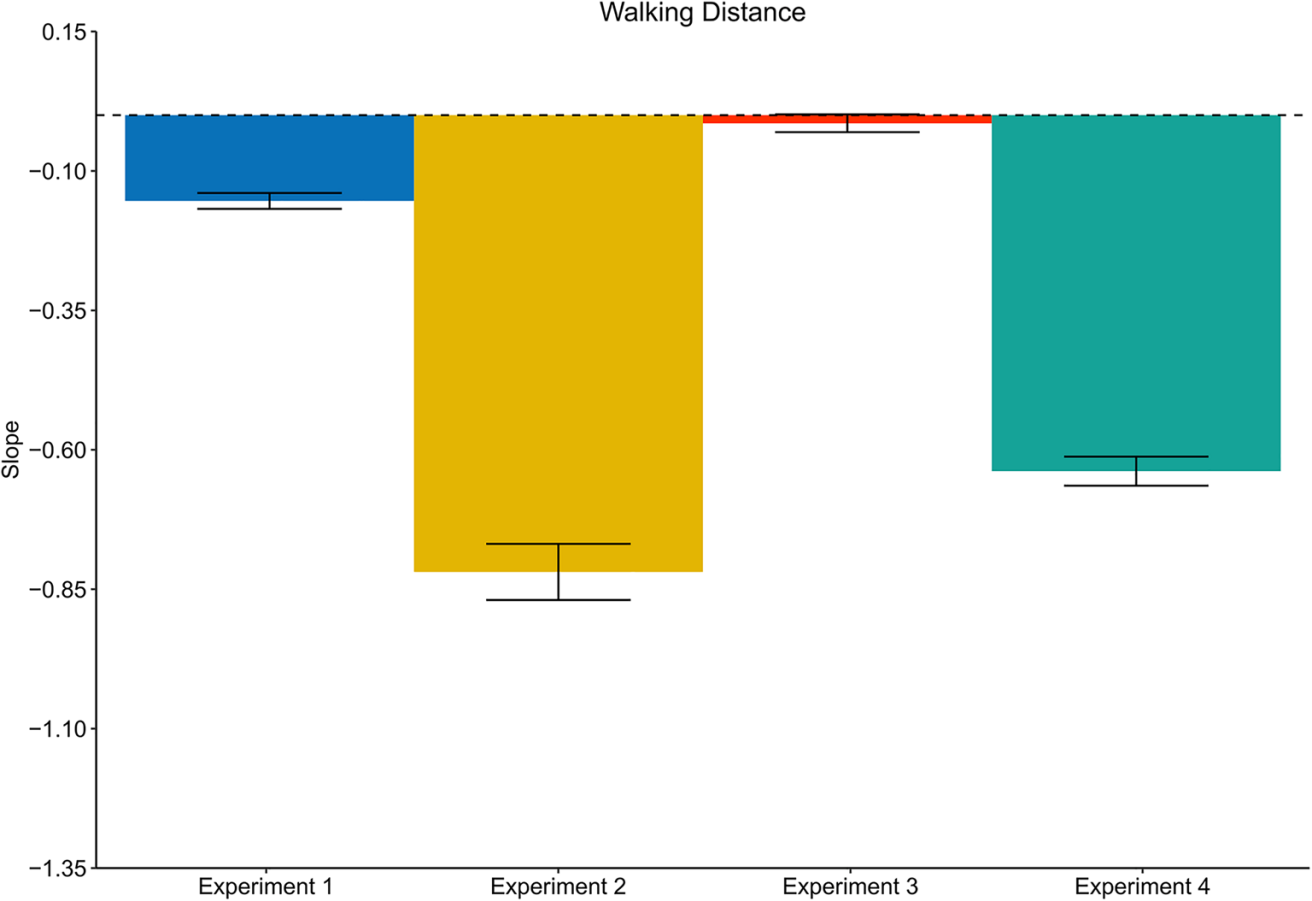
Average slopes representing the judged to distance ratio of walking distance judgements trials as a function of the translation gain for all experiments. Error bars represent the SEM. Experiment 1: physical walking with optic flow; Experiment 2: artificial walking with optic flow; Experiment 3: physical walking without optic flow; Experiment 4: physical walking with optic flow until prompted to stop.

Like in Experiment 1, the analysis did not reveal evidence for distance compression during the walking task (M = 1.02, SD = 0.06) (t(19) = 1.09, p = 0.29, *d* = 0.24), but significant distance compression for visual judgements (M = 0.93, SD = 0.12), (t(19) = − 2.67, p < 0.05, *d* = 0.60). We did not observe any serial dependencies between walking distances in trial n_w_ and the perturbed terminal feedback of trial n_W_ − 1 (Slope: M = − 0.01, SD = 0.08), (t(19) =  − 0.74, p = 0.47, *d* = 0.017) (Figs. 5 and 6).

**Figure 7:**
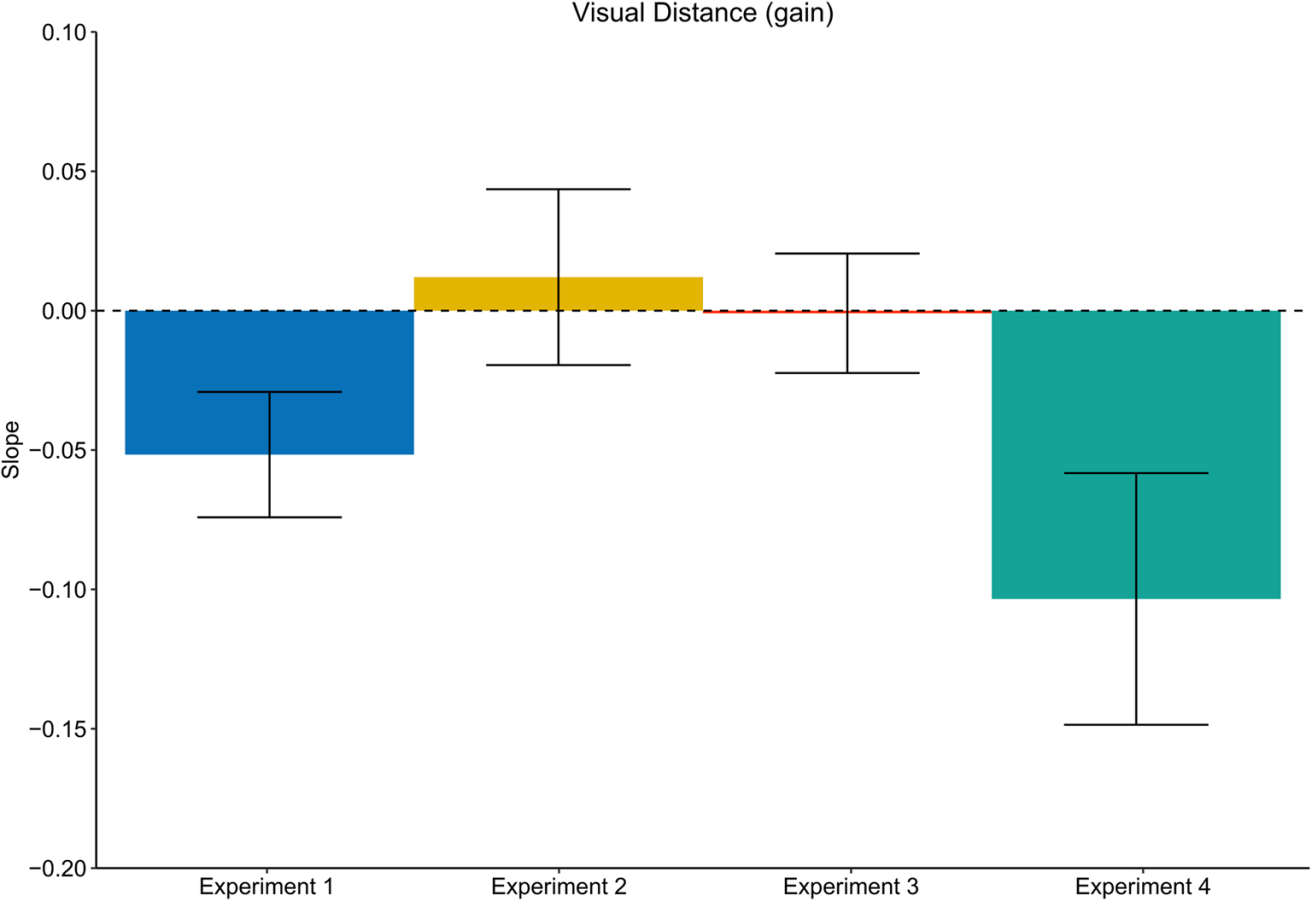
Average slopes representing the judged to distance ratio of visual judgement trials as a function of the translation gain in trial n_w_ − 1 for all experiments. Error bars represent the SEM. Experiment 1: physical walking with optic flow; Experiment 2: artificial walking with optic flow; Experiment 3: physical walking without optic flow; Experiment 4: physical walking with optic flow until prompted to stop.

Given the lack of recalibration effects on walking distance estimates, we expected not to see any effects on visual judgements. In line with this reasoning, the analysis did not provide evidence for serial dependencies between the walking trial n_W_ − 1 on visual distance judgements, neither as a function of the translation gain (Slope: M = − 9.3 × 10^–5^, SD = 0.10), (t(19) =  − 0.04, p = 0.97, *d* = 0.01) (Figs. 5 and 7) nor as a function of the travel distance in trial n_W_ − 1. (Slope: M = 8.0 × 10^–6^, SD = 0.001), (t(19) = 0.03, p = 0.98, *d* = 0.01) (Figs. 5 and 9).

As before, we observed a strong positive regression of the target error as a function of the translation gain (Slope: M = 237.40, SD = 34.40) which was significant different from zero (t(19) = 30.86, p < 0.001, *d* = 6.90) (Figs. S3, S11). However, again, the analysis did not provide evidence for serial dependencies of the target error at the end trial n_W_ − 1 on visual distance judgements in trial n_v_ (Slope: M = − 2.75 × 10^–5^, SD = 6.13 × 10^–4^), (t(19) =  − 0.20, p = 0.84, *d* = 0.04) (Figs. S3, S12).

In Experiment 3, we did not observe a significant effect of the error feedback at the end of trial n_W_ − 1 on the maximum walking speed in the following walking trial n_w_ (t(19) =  − 1.80, p = 0.09, *d* = 0.40) (Figs. S7, S9).

Consequently, the maximum walking speed (t(19) = 0.85, p = 0.41, *d* = 0.19) of trial n_W_ − 1 had no significant effect on visual judgements (Fig. S7).

### Experiment 4

In Experiment 4, we aimed to manipulate the travel distances directly. Generally, the procedure was identical to Experiment 1. However, the walking task did not involve any distance estimates, but participants walked until they heard a sound, indicating them to stop. The sound was presented when participants reached the visual target location. Again, a translation gain was used to change the ratio between visual and physical motion. As a result, the experimental manipulation of Experiment 4 ensured that the visual distance participants traveled was almost identical between trials, while the physical distance varied with the translation gain on a trial-by-trial basis.

The analysis revealed a significant compression of visual judgements (M = 0.89, SD = 0.18), (t(19) = 2.58, p < 0.05, *d* = 0.58). We found a significant distance compression for walking distances (M = 0.95, SD = 0.002), (t(19) =  − 115.88, p < 0.001, *d* = 25.9). However, instead of a perceptual effect, the distance compression was the result of the mechanism to detect when participants reached the target location. An invisible collision volume, which was used to detect when the Vive Tracker reached the target location, triggered the sound a few centimeters earlier as intended, resulting in a small but constant reduction of the physical travel distance.

**Figure 8:**
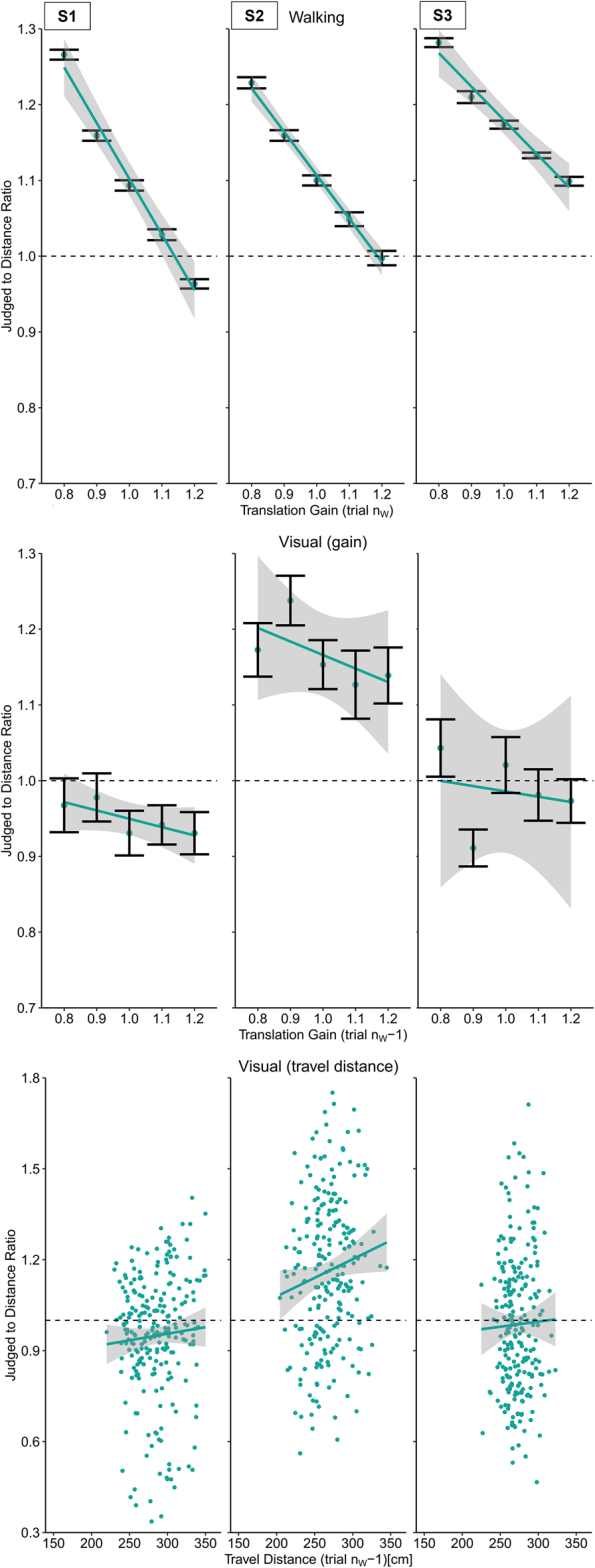
Results of Experiment 4 (physical walking until prompted to stop with optic flow) for three example participants. Upper panel: Regression between the translation gain in trial n_W_ and judge to distance ratio for walking distance estimations in trial n_W_. Middle panel: Serial dependencies quantified by the regression between the translation gain in in trial n_w_ − 1 and visual distance judgements. Lower panel: Serial dependencies quantified by the regression between travel distance in trial n_w_ − 1 and visual distance judgements. Error bars represent the SEM and the grey areas represent the 95% confidence interval.

To verify the effectiveness of manipulating the stop signal, we analyzed the distance participants traveled until the sound was played in relation to the translation gain. The results were significant (Slope: M = − 0.97, SD = 0.01), (t(19) =  − 332.02, p < 0.001, d = 74.24) For subsequent calculations involving physical travel distances, we recalculated the distance using a different method. Instead of measuring the distance until the stop signal occurred, we defined the end location as the point where participants stopped walking, taking into account any delays between the signal and the actual stopping point. This was done by asking participants to press a button when they stopped walking. The analysis resulted in a significant negative slope (Slope: M = − 0.64, SD = 0.12), (t(19) =  − 24.53, p < 0.001, d = 5.48) (Figs. 6, 8).

Furthermore, replicating Experiment 1, we observed serial dependencies between the translation gain in trial n_W_ − 1 and subsequent visual distance estimates in trial n_V_ (Slope: M = − 0.11, SD = 0.2), (t(19) =  − 2.32, p < 0.05, *d* = 0.52) (Fig. 8 and 7), indicating that participants estimate egocentric distances to be shorter after exposure to a higher translation gain and vice versa.

**Figure 9:**
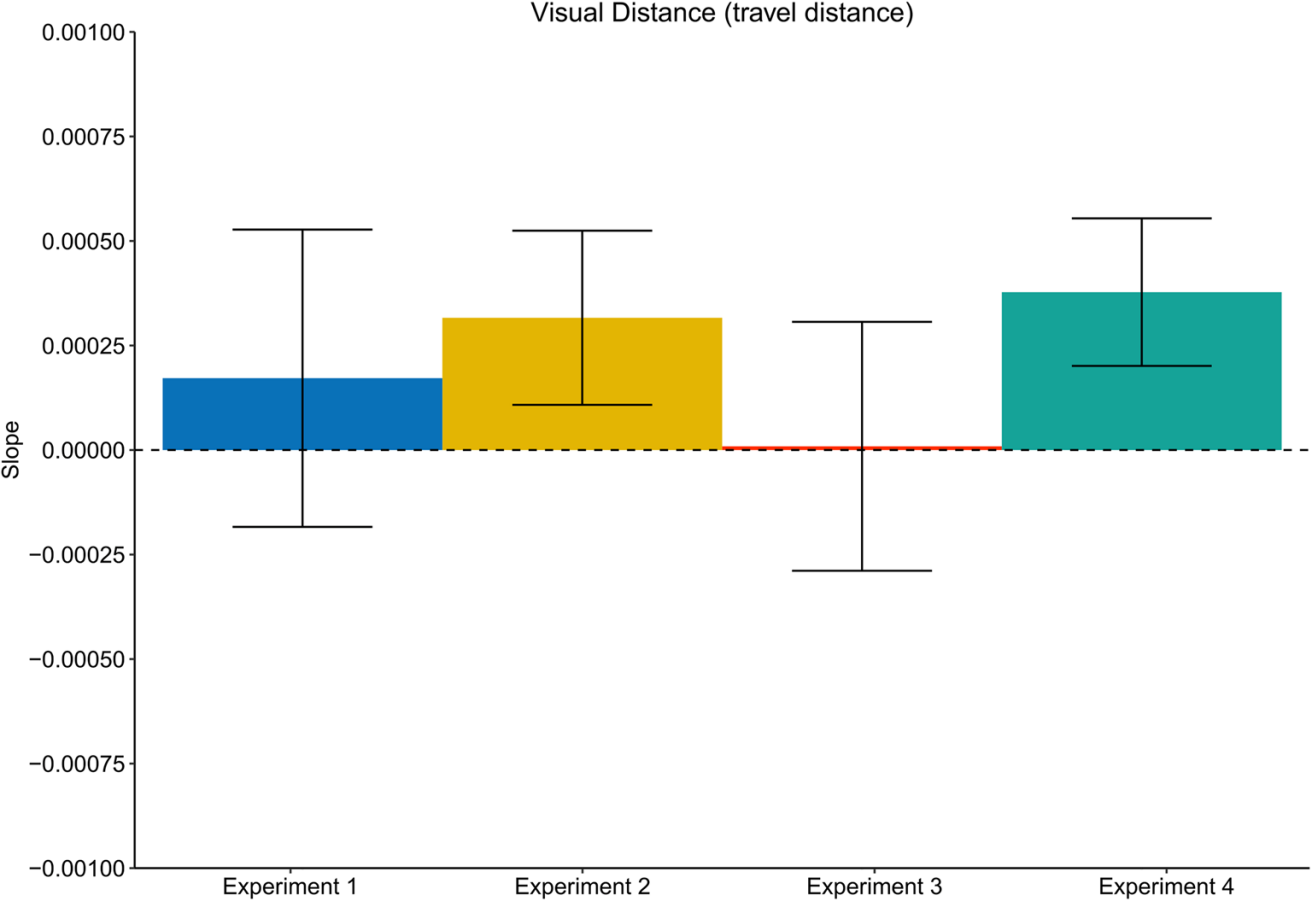
Average slopes representing the judged to distance ratio of visual judgement trials as a function of the travel distance in trial n_W_ − 1 for all experiments. Error bars represent the SEM. Experiment 1: physical walking with optic flow; Experiment 2: artificial walking with optic flow; Experiment 3: physical walking without optic flow; Experiment 4: physical walking with optic flow until prompted to stop.

We compared the slopes of the travel distances between the experiments in a Welch two-sample t-test, which resulted in a significantly stronger effect on the travel distances in Experiment 4 (t(20.6) = 56.3, p < 0.001, *d* = 17.81), indicating a steeper negative curve for the travel distances in Experiment 4. This suggest that the travel distances between the different translation gain levels were more diverse, i.e., that in Experiment 4 the difference in the distance traveled between the gain levels was larger than in Experiment 1. If the travel distance had caused the effect on the visual judgments in the Experiment 1, then the stronger modulation of travel distance in Experiment 4 should also result in stronger modulation of the visual judgements. To examine if the travel distance during in Experiment 1 and Experiment 4 was what drove the effects on visual judgements, we expected to see a significantly steeper negative slope for visual judgements in Experiment 4. However, a Welch two-sample t-test provided no evidence for this assumption (t(27.75) = 1.06, p = 0.30, *d* = 0.34). However, we found a small but significant effect of the physical walking distance of n_W_ − 1 on visual distance estimates in n_w_ (Slope: M = 3.8 × 10^–4^, SD = 8.0 × 10^–4^), (t(19) = 2.15, p < 0.05, *d* = 0.48) providing evidence that the travel distance modulated the visual judgements in the following trial, i.e., participants estimated the visual target to be farther away after walking a longer distance and vice versa (Figs. 8 and 9).

Again, the target error was analyzed, i.e., the distance between participants and the walking target at the moment of the button press. We observed a small positive regression of the target error as a function of the translation gain (Slope: M = 3.09, SD = 2.96) which was significant different from zero (t(19) = 4.67, p < 0.001, *d* = 4.04) (Figs. S4, S11). However, again, the analysis did not provide evidence for serial dependencies of the target error at the end trial n_W_ − 1 on visual distance judgements in trial n_v_ (Slope: M = − 2.75 × 10^–5^, SD = 6.13 × 10^–4^), (t(19) =  − 0.20, p = 0.84, *d* = 0.04) (Figs. S8, S12).

We could further replicate the effect of the translational gain on the maximum walking speed (t(19) =  − 2.73, p < 0.05, *d* = 0.61) (Figs. S8, S10). Similarly, we again did not find evidence for serial effects of the maximum walking speed on subsequent visual judgements (t(19) = 0.05, p = 0.95, *d* = 0.01) (Figs. S8, S9).

## Discussion

We examined if error signals caused by a mismatch between the visual and physical travel distance recalibrate visually perceived space in a serially dependent manner. Serial dependencies describe a weighted learning mechanism that biases perception by prior information of the recent past. Recently, serial dependencies between motoric information and visual perception have been reported^19,22–24^.

Previous research in rodents indicates that activity in primary visual cortex is modulated by self-motion information and information about the self-location^3–5^. Motor related error signals resulting from mismatches between visual and physical motion appear to have strong influence on the activity in primary visual cortex^6^. Fournier et al.^7^ showed that activity in primary visual cortex is modulated by the physically traveled distance. Idiothetic information, such as the travel distance, might be used to recalibrate visually perceived space.

Here, travel distances were manipulated by creating a mismatch between visual and physical motion speed during a walking distance estimation task. Previous research has repeatedly demonstrated that artificially increasing or decreasing the optic flow speed biases subsequent travel distances in blind walking tasks^9,10,27^. Participants typically estimate egocentric distances to be shorter in blind walking after being exposed to increased visual motion speed during walking, whereas decreasing visual motion has the opposite effect.

The results of Experiment 1 confirmed that the mismatch between predicted and sensed optic flow successfully manipulated the physical travel distances. Participants traveled farther when the visual motion speed was artificially reduced and shorter distances when visual motion speed of increased. If the brain uses the physical travel distance as a measure to recalibrate visual distances, we expected visual estimates of egocentric distances to be biased by the previously traveled distance in walking trial n_W_ − 1. We found no effects of the travel distance on visual distance judgement. Analyzing visual distance judgements as a function of the translation gain, on the other hand, provided clear evidence for serial dependencies between translation gain during walking trials and subsequent visual distance estimates. Participants estimated the visual target to be closer after walking trials with increased visual motion than after trials with reduced visual motion.

Importantly, the translation gain manipulation was only active during walking trials. For visual judgement the mapping between visual and physical motion of always 1:1. Furthermore, to minimize visual distance cues, visual judgements were conducted in a sparse ground plane environment. The separation of the environments together with the absence of any manipulation during visual judgements ensured that any serial effects observed must be caused during the walking task.

Hence, we examined additional potential factors that may have contributed to the observed serial effect. Instead of considering physical travel distance, we examined the effect of target error—the distance between the shifted walking target and the participant at the end of the trial. In a first step, to verify our manipulation of the translation gain, we observed a strong positive correlation between the translation gain and the target error. We also analyzed the relationship between visual distance judgements and target error in the previous trial but found no evidence to support this as a contributing factor. Additionally, we examined the effect of maximum walking speed on visual distance judgements. In line with previous research^28,29^, we observed an increase in maximum walking speed, when visual motion was reduced and vice versa. However, the analysis did not provide evidence, that the serial effects observed for visual judgements were affected by the maximum walking speed in trial n_W_ − 1.

In principle, changes in visual space could be driven purely visually by the experimental changes in optic flow^30^. We conducted a separate experiment in which stationary participants visually traveled the distance via thumb stick to isolate the role of optic flow. Body-based information, such as proprioceptive or vestibular cues, were not available in Experiment 2, and travel distances could only be estimated using visual information. We observed a greatly increased effect on travel distances. Importantly, we did not observe serial dependencies between the translation gain of walking trial n_W_ − 1 on visual distance estimates in trial n_v_, suggesting that a varying visual self-motion speed alone is not sufficient to calibrate visually perceived distances.

Experiment 3 aimed to examine to role body-based cues resulting from physically walking. Walking was performed in the ground plane environment by participants in the absence of optic flow. Instead of manipulated optic flow, participants in Experiment 3 received perturbed terminal feedback. We expected to observe serial dependencies of the manipulated terminal feedback of walking trial n_W_ − 1 on the travel distance in the following walking trial n_w_. Contrary to our hypothesis, we did not find any recalibration effects of travel distances. Consequently, we did also not observe serial effects on visual judgements.

This result is inconsistent with Mohler et al.^9^, who found improved distance estimates for both blind walking and verbal distance estimates after participants received veridical terminal feedback after blind walking. In contrast to Mohler et al.^9^, who provided veridical terminal feedback, in Experiment 3, the terminal feedback was randomly perturbed on a trial-by-trial basis. Instead of using the terminal feedback of the previous trial to correct the distance estimate of the current trial, participants might instead just have calibrated their distance estimates based on the mean feedback.

Additionally, serial dependencies have been found to vary depending on the context^33^ and changing environments might have decreased the size of the serial dependencies. Fischer et al., 2020 showed that serial dependencies in vision are context dependent. However, the requirements of the experimental setup, i.e., providing optic flow during walking and removing landmarks in localization trials, made the context switch necessary. In all experiment, except of Experiment 3, participants switched from a complex environment (trial n_W_ − 1) to a sparse ground plane environment (trial n_v_). By switching to a sparse environment, we aimed to only provide minimal depth information that could be used to correct for serial biases. However, in Experiment 3, we switched from a simple to a complex environment, which provided new information that could have been used to override and eliminate serial biases from trial n_W_ − 1. Therefore, a more sensitive design might have been to conduct both walking and visual judgments in the simple ground plane environment.

Given the absence of an effect of the terminal feedback on travel distances in Experiment 3, it is not surprising that we did not observe any effect of the terminal feedback on visual distance judgements. However, this result suggests that the serial effects on visual judgements, observed in Experiment 1, depend on differences in the travel distance.

Hence, another experiment was conducted to examine the role of the travel distance in visual judgements more directly. In Experiment 4 travel distances were directly manipulated by having participants to walk until they heard a tone, while applying the translation gain. This procedure resulted in a strong effect on the travel distances, which differed significantly from the effects observed in Experiment 1.

We found serial dependencies between the translation gain in trial n_W_ − 1 and visual distance judgments in trial n_v_, replicating the effects observed in Experiment 1.

Furthermore, the experiment resulted in a small but significant effect of the travel distance in the prior walking trial on visual distance judgements. A possible explanation might be that the modulation of travel distances was weaker in Experiment 1, resulting in only minor recalibration and that the experiment design was not sensitive enough to detect such small effects. In contrast, the manipulation in Experiment 4 resulted in stronger modulations of travel distances, which may have resulted in stronger recalibration of visual distances.

Importantly, the effect of travel distances on visual judgements was small, possibly accounting for only a minor portion of the effect observed for translation gain on visual judgments. In fact, the effect is so small that it is questionable whether it is a valid effect at all. However, even if the effect is valid and replicate-able, the small effects size suggests that the travel distance only accounts for a small portion of the visual recalibration effects and that other idiothetic signals are also used to recalibrate visual space, such as the number of steps it takes to reach a target location. Previous research has demonstrated that manipulation of the optic flow speed result in immediate corrections of the leg movement^29^. Future research should include tracking of the feet into their research to get detailed information about foot and leg movement parameters, such as the step length or step frequency.

The results of Experiment 4 contradict Kunz et al.^15^. In their study, participants walked until an auditory signal told them to stop, either twice as fast or half as fast as the physical motion. While the results of a blind walking task clearly showed visual motor recalibration effects, no transfer to size judgements was observed. However, participants did not receive feedback on the distance traveled, so there was no conflict between expected and actual walking distance. In Experiment 4, a visual target was presented before and after walking. The feedback provided by the target could have provided the error signal to recalibrate perceived space. Yet, analyzing visual distance judgements as a function of target error in trial n_W_ − 1 did not provide evidence that the error feedback provided by the walking target had serial effects on egocentric perceived distances in Experiment 4. Future research should examine if serial effects on visual distance judgements remain without a target shown after the walking trial.

Kunz et al.^15^ argue that effects of locomotion on visual distance estimates found in previous research (see for example Kelly et al.^16^) might be the result of cognitive corrections. Hence, instead of a perceptual effect, participants might have learned during trial n_W_ − 1 that the walking target was farther or closer than it appeared before, and a cognitive correction could be applied to the visual judgements afterwards. The current study cannot rule out that the effects observed for visual distance judgements are the result of cognitive corrections rather or a perceptual effect. In fact, there is also some controversy about whether serial dependencies occur on a perceptual or on a decision level^35,36^.

Taken together, the experiment reported in this study show that walking through an environment biases visually perceived distances in a serially dependent manner. However, the serial effects are not observed when participants only receive visual information of self-motion, without physically walking, indicating that information gained from locomotion is required to observe recalibration effects. Manipulated terminal feedback after physical walking in the absence of optic flow did not results in recalibration effects of travel distances. Given that we did not observe differences in the walking task between the different gains in Experiment 3 suggest that differences of the travel distance or related parameters are necessary for serial effects on visual judgements.

Kelly et al. (2013) argued that walking interactions in VR causes a rescaling of perceived space, which should affect all kinds of distance estimates. They suggest a perceptual learning mechanism to account for e.g., incorrect depth cues or missing cues in VR. Walking interaction could result in a learning process in which perceptual cues are weighted according to their reliability. However, in the present study we show transfer of changes of physical travel distances on visual depth estimates between two completely different virtual environments, thus arguing for recalibration. Furthermore, the rescaling account by Kelly proposes a shared resource underlying both walking distance estimates and visual distance estimates, which would predict a full transfer from walking to visual judgements. However, in the present study, effects on visual distance estimates were only a fraction of effects on walking distance estimates.

In conclusion, the results of the present study show that egocentric depth perception is embodied and recalibrated by walking through an environment. Linking the perception of depth directly to idiothetic signals, such as the travel distance, provides a computationally efficient means of calibration. Since the sensorimotor system constantly monitors movement performance, these signals can be used with the only extra neural cost of being fed back to visual areas.

## Limitations

Due to limited space in the laboratory, the walking target was always presented at a distance of 2.5 m. In principle, participants could have counted their steps, to hit the initial shown target location. However, since we observe strong effects of the translational gain on travel distances, this might at worst have reduced the effects. Future studies should test if the effects further increase after longer walking distances.

In the present study we did not attach motion trackers to the feet, which did not allow us to determine the impact of the experimental manipulations on the stride length or the number of steps. Especially the latter could serve as a measure to recalibrate visual distances.

Lastly, here, we used a tethered HMD. The cable of the HTC Vive is about 5 m long, which limits the maximum walking range. While the experimental setup was carefully arranged that all possible locations a participant could walk to, where within the range of the cable, especially for longer distances, a slight pulling of the cable could not be avoided and might have served as a distance cue. Future studies should instead be conducted using wireless HMDs, such as the HTC Vive Pro together with the available wireless-adapter or a standalone HMD such as Oculus Quest 2 or Pico Neo 3.

## Experiment 1

### Participants

20 participants (17 females, ages 19–30, 1 left-handed) took part in Experiment 1. Each participant completed 4 sessions of the experiment, each at least separated by half an hour. All participants had normal or corrected-to normal vision. All participants gave their written informed consent prior to participation and subsequently received either monetary compensation or course credits. All experiments were approved by the ethics committee of the Faculty of Mathematics and Natural Sciences of the Heinrich-Heine-University Düsseldorf, Germany and the study procedures were in line with the declaration of Helsinki.

### Apparatus

Stimuli were delivered by the HTC Vive Pro Eye^37^. The HMD presents stimuli on two low-persistence organic light-emitting diode (OLED) displays with a resolution of 1440 × 1600 pixels per eye and a refresh rate of 90 Hz. Participants also received a pair of Valve Index controllers^38^. Head and hand movements were tracked via the HMD and controllers with the SteamVR 2.0 tracking system using 4 base stations. Additionally, a Vive Tracker 2.0 was attached to the waist. Motion tracking data of the HMD, controllers and Vive tracker was sampled at 90 Hz. Furthermore, the SteamVR skeletal input system was used to allow the finger curls to be tracked. The hand and finger movement were represented using gloves instead of rendered hands. The virtual environment was rendered using a custom-made program created in the Unreal Engine 4.25^39^. The SRanipal eye tracking calibration software was used prior to each experimental session to ensure a correct fit of the HMD and for adjusting the inter-pupillary distance^37^.

### Task and stimuli

The virtual environment consisted of a rectangular room of 10.5 m × 12 m (Fig. 1, upper panel and Fig. 2). Adjacent to the two longer walls were two identical livings rooms, containing some furniture, such as a couch and table. The furniture had the same scale as its real-world counter parts. The living rooms served as a size reference in the otherwise rather monotonous environment. However, participants were asked to look straight forward during walking and as a result the living rooms were outside of the HMD’s field of view of about 110° (approximately 90° per eye)^40^ (see Fig. 2). Hence, the living rooms were not visible during the task itself.

Furthermore, we aimed to render the environment in a realistic manner, which has been found to reduce distance compression effects VR in verbal distance estimation tasks and judgements of object size^14^. Realistic rendering is also associated with a stronger experience of presence in VR, i.e., the feeling of *being there* in the virtual environment rather than physical reality^41,42^, which is also associated with reduced distance compression effects in VR^43,44^.

During the experiments, participants received task instructions written on two displays, each attached to one of the shorter walls.

Previous research has shown that the distance estimates in virtual environments are affected by the virtual eye-height^45^. Here, the virtual floor of both environments was calibrated to align with physical floor of the laboratory. Furthermore, the tracking origin of the first-person camera was set to floor level, which ensured that the eye height in the virtual environment was identical to the participant’s real eye height.

During the experiment, SteamVR’s chaperone system was deactivated. The chaperone system is used to display the boundaries of the physical space. As soon as the HMD or one of the controllers approaches a boundary, the chaperone system automatically provides visual cues notifying the participant that they are close to a boundary. Instead, here we used a buffer area, which extended about 2 m beyond the target locations, to ensure safety during the experiment.

To prevent participants from using learned distance cues or landmarks of the indoor environment, visual distance judgements were conducted in a within a monotonous ground plane environment. The environment consisted of an optically infinite quadratic plain (0.4 km^2^) without any objects and or textures, which could give information about distances. The environment further showed a night-sky showing some stars and a few clouds (Fig. 1 middle panel). The sky-sphere containing the night-sky texture had a diameter of 32.8 km.

### Procedure

Each experimental session contained an n-back trial structure in which walking trials (trial n_W_ − 1) alternated with visual judgement trials (trial n_v_). To start a walking trial, participants had to stand on a red positional marker. After pressing the trigger button of the right controller, a blue fixation sphere appeared 1.25 m in front of the participants at a height of 1.25 m (diameter: 5 cm). The fixation period was included to ensure that participants are looking into the direction where the target appeared. To avoid visual references when the walking target was shown, the fixation sphere disappeared after 1 s. However, participants were instructed to keep their gaze along the same direction until the target was presented.

After a random interval between 1.0 and 1.5 s, the target location was highlighted with a flat red circle (diameter: 50 cm) for 200 ms on the floor at 2.5 m. The distance of 2.5 m was fixed in all trials.

Participants were then instructed to walk to the remembered target location. Once they believed to have reached the correct location, participants pressed a button to indicate the end of the walking. Then, the walking target reappeared. If participants missed the target location, they were asked to correct their position. The target contained a small red arrow, indicating in which direction participants should turn around after walking. This was necessary to prevent the HMD’s cable from tangling. To account for the limited cable length of the HMD of about 5 m, the computer running the experiments, was placed on the side of the walking track, in the middle between the start and target stimulus locations, thus avoiding any pulling of the cable.

Each walking trial was followed by a visual distance judgement trial. Participants were asked to turn around and to fixate another blue fixation sphere for 1 s. After the fixation period, the sphere and the entire room disappeared, and participants found themselves in a ground plane environment consisting of an endless gray floor and a dark night sky, showing some stars and clouds. The environment was designed to minimize available monocular distance cues, to ensure that the distance judgements were not biased by the rich spatial cues provided by the indoor environment. Again, after a random interval between 1.0 and 1.5 s, the target, a flat green circle, appeared for 33 ms on the ground. The target could appear at of six different distances from the start position (0.8 m, 1.0 m, 1.2 m, 1.4 m, 1.6 m, 1.8 m). Participants were asked to estimate the distance in centimeters between themselves and the green circle. The possible combinations of target distances in the visual judgement trial n_V_ and the translation gain of the walking trial n_W_ − 1 were randomized but counterbalanced to ensure that each combination was shown equally often.

The distance estimates were entered through a virtual numeric keypad (Fig. 1, lower panel). The table was only visible after the green target was presented and appeared behind the participants, to not interfere with the task. The table also contained a small screen to display the distance estimates and, during training trials, to give feedback about the task performance. Participants were asked to enter their estimate in centimeters. After typing in the number, participants pressed the big orange button, which was left to the numeric keypad, to confirm their estimate. Alternatively, participants could correct their estimate by using the delete button and type in a new estimate. Once the estimate was entered, the indoor environment reappeared, and the next walking trial was started.

Each session started with 14 trainings trials, which served for familiarization with virtual environment and the tasks. During training trials, participants received feedback about their visual distance estimates on the display placed next to the numeric keypad. A negative number indicated that the distance was underestimated, and a positive value indicated an overestimation. After the training trials, participants completed 60 test trials in each session. In contrast to the training, no feedback about the distance estimates was given at the end of the trial. A single session of the experiment lasted for about 30–50 min. In total, 296 trials were conducted across the four sessions, of which 240 trials were test trials, i.e., 240 walking distance estimates and 240 visual judgements.

### Experimental manipulation

During the walking trials the ratio between physical and virtual distance was altered, by using a 2D translation gain, which changes the participant’s movement along the horizontal plane^25,27^. A translation gain of 1.0 corresponds to an isometric mapping, i.e., the virtually walked distance corresponds 1:1 with physical distance. A translation gain greater than 1.0 corresponds to an increased optic flow, whereas a translation gain smaller than 1.0 corresponds to a reduced optic flow. The translation gain was achieved by moving the virtual environment including all objects relative to the participant’s movement. For example, a gain of 0.9 would result in the environment moving in the same direction as the participant at 10% of the participant’s walking speed, resulting in reduced visual motion. In trials with a translation gain of e.g., 1.1, the virtual environment moves with 10% of the participant’s walking speed, but in the opposite direction, resulting in increased optic flow.

During training trials, a gain of 1.0 was applied, i.e., the movement was isometrically mapped to the VE. During walking test trials, gain values ranged between 0.8 and 1.2 in steps of 0.1. The order of the gain values was randomized but counterbalanced, so that each gain value was tested 12 times per session, i.e., 48 repetition per gain after all 4 sessions. The translation gain was only applied during walking, i.e., between target-onset and the button press, indicating the end of walking. During visual judgement trials the translation gain was set to 1.0.

Since the translation gain manipulation resulted in a shift of the virtual relative the real environment, we had to set back the room environment to its original position after the visual judgement, to prevent a drift of the environment over time.

### Analysis

The free statistical software R^46^ and RStudio^47^, the packages doBy^48^ and LSR^49^ were used to analyze the behavioral data. Plot were generated using ggplot2^50^.

In a first step, the walking distance for each trial and participant was calculated. Location measurements were derived from the tracking data obtained with the Vive Tracker, which was attached to the participants waist.

Next, the ratios of the judged-to-actual distance for both walking distance estimates, and visual judgements were calculated for each trial. For walking trials, a ratio above 1.0 indicates that the target location was overshot and vice versa. For visual judgements, a ratio above 1.0 indicates an over estimation of the distance and vice versa. Distances were only calculated along the horizontal plane, while ignoring movement along the y-axis. We then estimated linear regressions between the translation gain and the travel distances for every participant. The resulting slopes were used to quantify the effect of the optic flow speed on the travel distance. T-tests were conducted to test whether the slopes are different from zero. Similarly, linear regressions between the translational gain and visual distance judgements as well as between the travel distances and visual distance judgements were calculated for each participant. The resulting slopes were then used to quantify the magnitude of the serial dependencies and t-tests were used to determine if the slopes are different from zero.

Training trials were not included in the analysis.

Furthermore, in a few instances, participants pressed the button, indicating the end of the walking trial, already at the start of the trial before walking to the target location. Hence, for those trials, no information about the traveled distance was available. Hence, each trial in which the walking distance did not exceed at least 150 cm was excluded from the analysis.

Visual judgements which deviated by 2.5 absolute deviations of the median, were seen as outliers and excluded from the analysis^51^. Across participants, 4.5% of trials were defined as outliers and removed from the analysis of Experiment 1.

Afterwards we analyzed maximum walking speed. The walking interval was defined as the first time the walking speed exceeded 0.5 m/s until the last time is fell below 0.5 m/s.

The outlier correction was identical to the one used above but we additionally excluded trials in which participant did not exceed a minimum speed of 0.5 m/s. Furthermore, trials in which the maximum speed exceeded 2.77 m/s, were excluded from the analysis. On average, the maximum speed across participants was at 1.15 m/s.

## Experiment 2

### Participants

20 participants (13 females, age 18–31 years, 2 left-handed) took part in Experiment 2. Each participant completed 2 sessions of the experiment, each separated by at least half an hour. All participants had normal or corrected-to normal vision. All participants gave their written informed consent prior to participation and subsequently received either monetary compensation or course credits. All experiments were approved by the ethics committee of the Faculty of Mathematics and Natural Sciences of the Heinrich-Heine-University Düsseldorf, Germany and the study procedures were in line with the declaration of Helsinki.

### Apparatus

The experimental setup was identical to Experiment 1.

### Task and stimuli

The tasks and stimuli were essentially identical to Experiment 1. The only difference was the method used for locomotion. Instead of physical overground walking, participants only walked virtually using the analog stick of the left controller. The analog stick was programmed that forward, backward, and sideward movement was possible by pressing the stick in the respective direction. The heading direction was based on the Vive Tracker’s forward direction. The movement speed could be controlled by the amount by which the thumb stick was pressed forward, with a maximum of 1.4 m/s when the translation gain was set to 1.0. Again, the translation gain was achieved by moving the VE relative to the participants movement in trials with a gain different from 1.0. Participants remained physically standing during the entire experiment.

### Procedure

The procedure was essentially identical to Experiment 1. Again, each trial started with the walking task and was followed by the verbal distance estimation task. However, each participant only completed 2 Session, each consisting of 14 training trials, followed by 60 test trials, resulting in 120 test trials per participant. The number of trials per participant was reduced, since this kind of artificial locomotion increases the chance that participants might suffer from motion sickness^52–54^. To ensure that the reduced number of trials did not result in an underpowered design, we first recalculated the analysis of Experiment 1, but only for the first two sessions, to see if the observed effects survive. Indeed, both the slopes representing the judged-to-distance ratio for walking trials (t(19) =  − 10.58, p = 2.1 × 10^–9^, *d* = 2.366) as well as for visual judgements as a function of the translation gain (t(19) =  − 2.598, p = 0.018, *d* = 0.581) remained significant.

Again, the translational gain was set to 1.0 during training, which then also served to familiarize participants with the medium movement speed.

We did not quantify simulator sickness by e.g., using a questionnaire, but participants were told beforehand that they should stop the experiment if they feel symptoms. In total, 3 participants did not finish the experiment. Hence, 3 additional participants were tested.

### Analysis

The analysis was identical to Experiment 1.

Across all participants of Experiment 2, 2.25% of all trials were defined as outliers and removed from the analysis. Since the maximum possible movement speed was set to value of about 1.4 m/s, the cutoff for the maximum speed was reduced 1.6 m/s for the analysis of the walking speed, resulting in 16 excluded trials.

## Experiment 3

### Participants

20 participants (10 females, age 19–33, 3 left-handed) took part in Experiment 3. Each participant completed 4 sessions of the experiment, each at least separated by half an hour. All participants had normal or corrected-to normal vision. All participants gave their written informed consent prior to participation and subsequently received either monetary compensation or course credits. All experiments were approved by the ethics committee of the Faculty of Mathematics and Natural Sciences of the Heinrich-Heine-University Düsseldorf, Germany and the study procedures were in line with the declaration of Helsinki.

### Apparatus

The experimental setup was identical to Experiment 1.

### Task and stimuli

For Experiment 3, we switched the environment in which the tasks were completed. To eliminate optic flow during walking, the walking task was conducted within the ground plane environment and the verbal distance estimation task was conducted in the room environment. Consequently, participants did not receive perturbated feedback during walking. After participants indicated via button press that they believed to be at the target location, the room environment including target stimulus reappeared. However, the environment as well as the target stimulus were shifted along the walking direction, to mimic the perturbations resulting from the translation gain of Experiment 1.

### Procedure

The procedure was identical to Experiment 1. Again, each trial started with the walking task and was followed by the verbal distance estimation task. Participants completed 14 training trials, followed by 60 test trials in each session, resulting in 240 test trials per participant.

### Analysis

The analysis was essentially identical to Experiment 1. However, since in Experiment 2 the walking task was changed to walking without optic flow, participants only received feedback about their walking performance at the end of the trial. Accordingly, we estimated a linear regression between the translation gain of trial n_W_ − 1 and the travel distance of trial n_W_. To analyze the first test trial, we used the last training trial as n_W_ − 1.

Across all participants, 4.46% of the were defined as outliers and removed from the analysis. For the analysis of the walking speed, no additional trials were excluded.

## Experiment 4

20 participants (12 females, age 20–31, 1 left-handed) took part in Experiment 4. Each participant completed 4 sessions of the experiment, each at least separated by half an hour. All participants had normal or corrected-to normal vision. All participants gave their written informed consent prior to participation and subsequently received either monetary compensation or course credits. All experiments were approved by the ethics committee of the Faculty of Mathematics and Natural Sciences of the Heinrich-Heine-University Düsseldorf, Germany and the study procedures were in line with the declaration of Helsinki.

### Apparatus

The experimental setup was identical to Experiment 1.

### Task and stimuli

The stimuli and the distance judgement task were identical to Experiment 1. However, the walking task of Experiment 4 did not involve any distance estimations, but participants walked until they heard a ringing sound (duration about 800 ms) indicating to stop. Furthermore, like in the previous experiments, participants had to indicate that they stopped walking by button press. This was done to account for any delays between the stop signal and participants stopping to move. With the button press, the target reappeared and the translation gain was set to 1.0.

Apart from this change, the physical target distance remained the same of 2.5 m. Like in the previous experiments, the translation gain was applied during walking. Hence, here the walking distance was directly manipulated by the gain. This direct manipulation allowed us to test the effect of the walking distance on visual distance judgements observed in Experiment 1.

### Procedure

The procedure was identical to Experiment 1. Again, each trial started with the walking task and was followed by the verbal distance estimation task. Participants completed 14 training trials, followed by 60 test trials in each session, resulting in 240 test trials per participant.

### Analysis

The analysis was identical to Experiment 1.

Across all participants, 5.29% of trials were defined as outliers and removed from the analysis.

## Supplementary Information

Supplementary Figures.

## Data Availability

For data analysis, we used custom built scripts in R. The data that support the findings of this study as well as analysis scripts and built version of the experiments are available at https://osf.io/pvzx9/.
