# Supplementary material for: Serial dependencies between locomotion and visual space
Source: Sci Rep. 2023 Feb 27;13:3302. doi: 10.1038/s41598-023-30265-z (PMC9970965; doi:10.1038/s41598-023-30265-z)
Supplement: Supplementary file 1 — Supplementary Figures. [file 41598_2023_30265_MOESM1_ESM.docx]

**Step by step - Serial dependencies between locomotion and visual space – Supplemental Information**

**Michael Wiesing^1^, Eckart Zimmermann^1^**

^1^ Institute for Experimental Psychology, Heinrich Heine University Duesseldorf, Germany


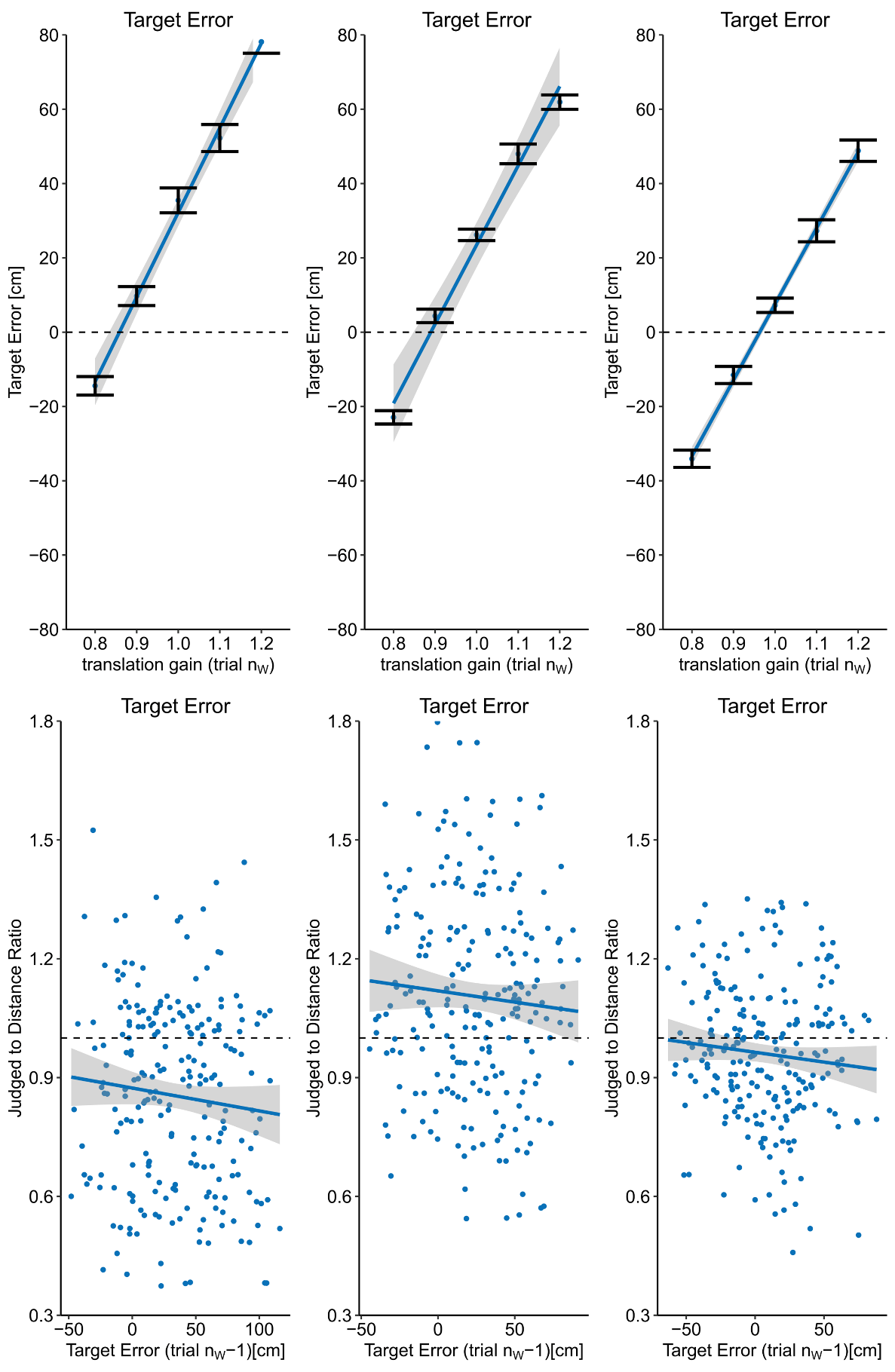


Figure S1 Target error results of Experiment 1 for three example participants. Upper panel: Regression between the translation gain in trial n_W_ and the target error for walking distance estimations in trial n_W_. Lower panel: Serial dependencies quantified by the regression between target error in trial n_w_ -1 and visual distance judgements. Error bars represent the SEM and the grey areas represent the 95% confidence interval.


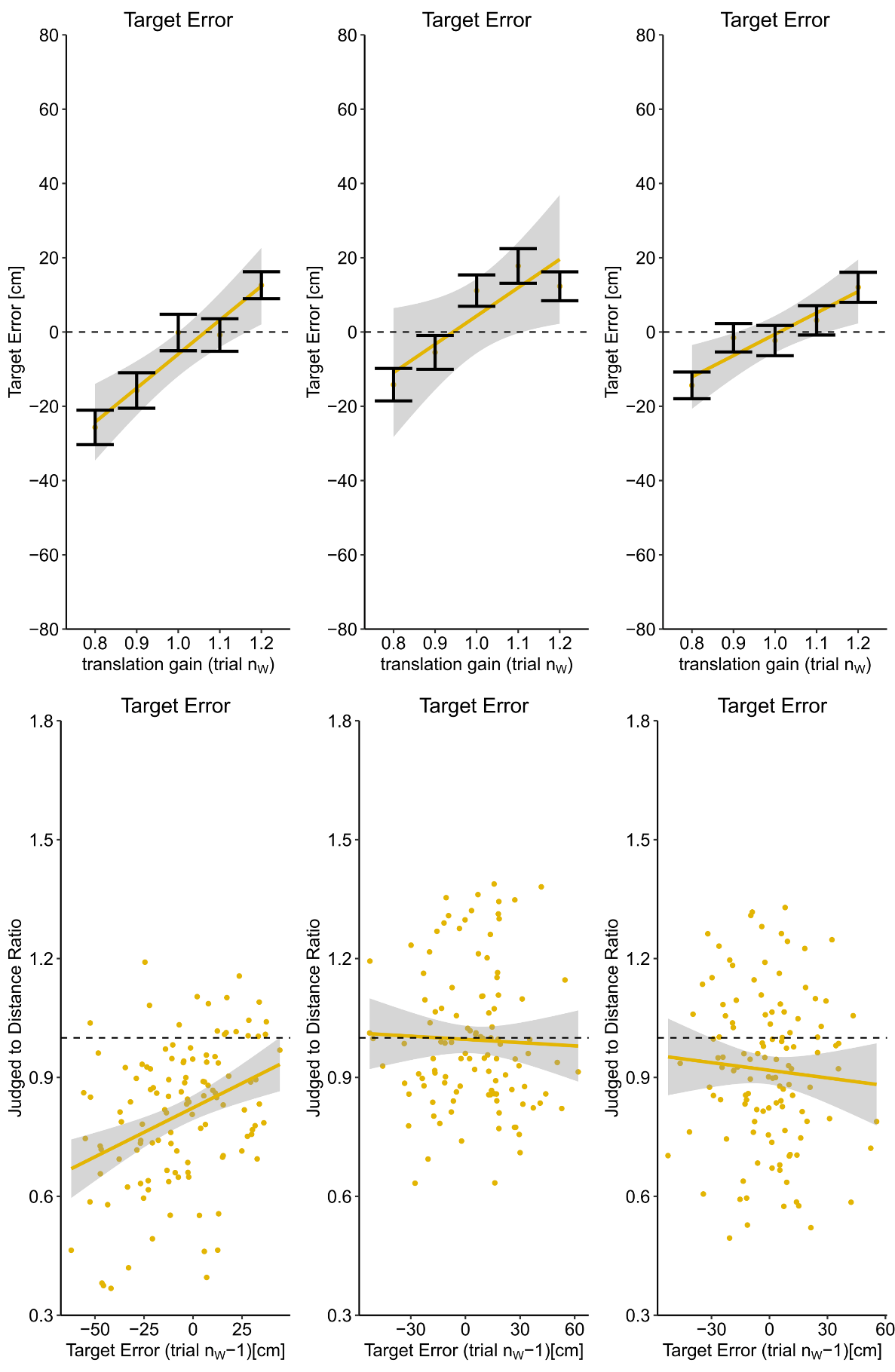


Figure S2 Target error results of Experiment 2 for three example participants. Upper panel: Regression between the translation gain in trial n_W_ and the target error for walking distance estimations in trial n_W_. Lower panel: Serial dependencies quantified by the regression between target error in trial n_w_ -1 and visual distance judgements. Error bars represent the SEM and the grey areas represent the 95% confidence interval.


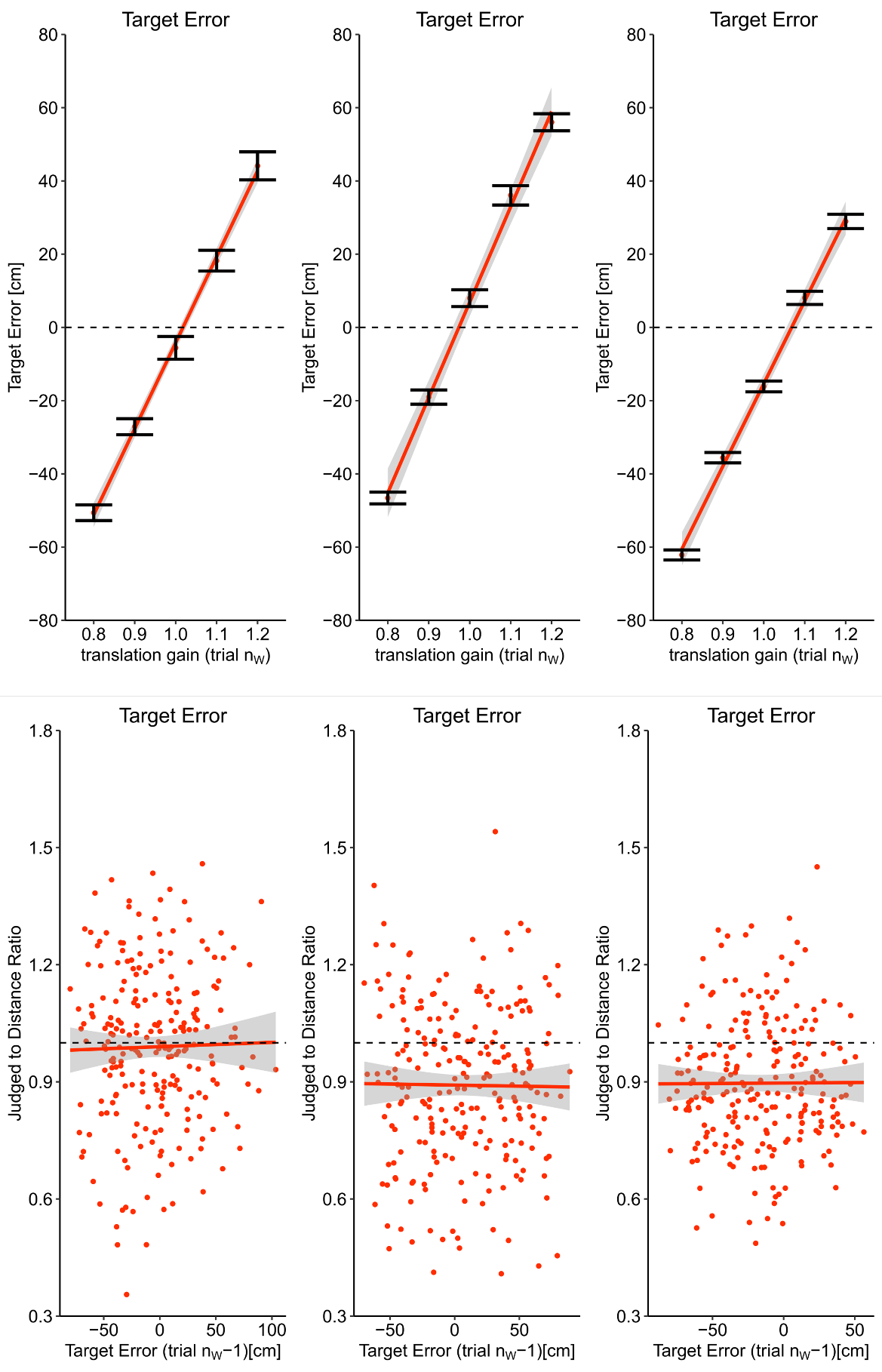


Figure S3 Target error results of Experiment 3for three example participants. Upper panel: Regression between the translation gain in trial n_W_ and the target error for walking distance estimations in trial n_W_. Lower panel: Serial dependencies quantified by the regression between target error in trial n_w_ -1 and visual distance judgements. Error bars represent the SEM and the grey areas represent the 95% confidence interval.


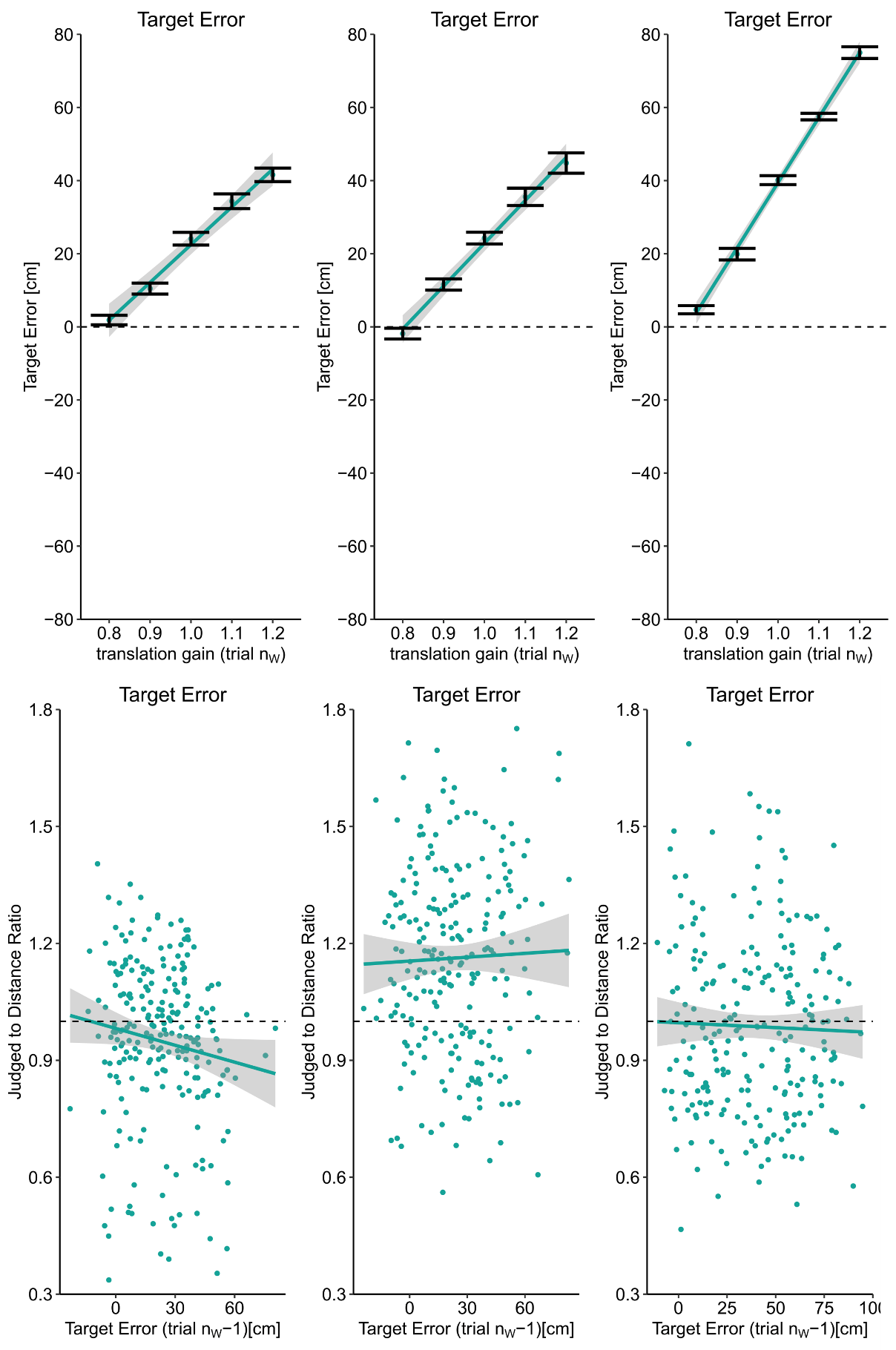


Figure S4 Target error results of Experiment 4 for three example participants. Upper panel: Regression between the translation gain in trial n_W_ and the target error for walking distance estimations in trial n_W_. Lower panel: Serial dependencies quantified by the regression between target error in trial n_w_ -1 and visual distance judgements. Error bars represent the SEM and the grey areas represent the 95% confidence interval.


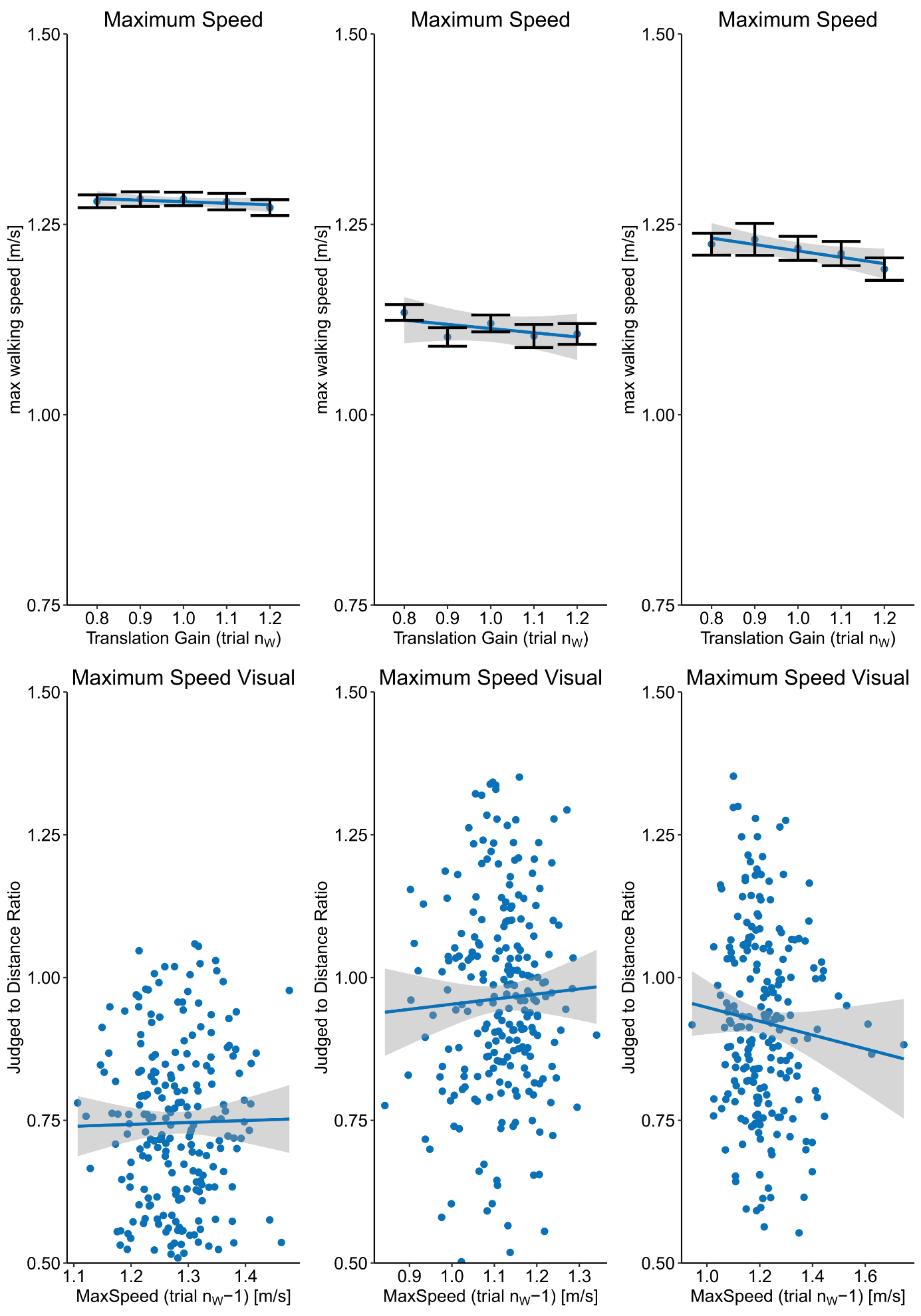


Figure S5 Maximum walking speed results of Experiment 1 for three example participants. Upper panel: Regression between the translation gain in trial n_W_ and maximum walking speed walking distance estimations in trial n_W_. Lower panel: Serial dependencies quantified by the regression between maximum walking speed trial n_w_ -1 and visual distance judgements. Error bars represent the SEM and the grey areas represent the 95% confidence interval.


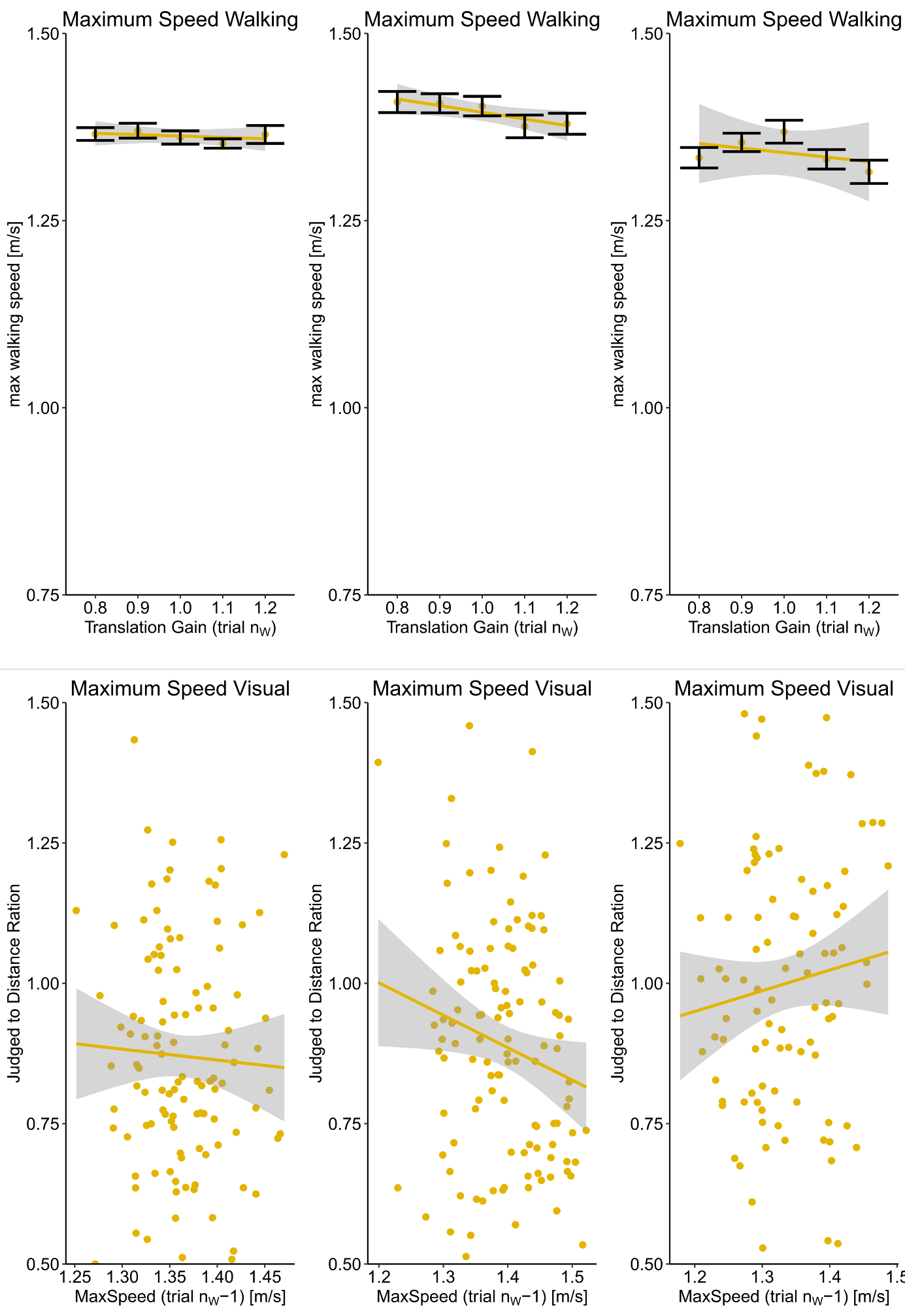


Figure S6 Maximum walking speed results of Experiment 2 for three example participants. Upper panel: Regression between the translation gain in trial n_W_ and maximum walking speed walking distance estimations in trial n_W_. Lower panel: Serial dependencies quantified by the regression between maximum walking speed trial n_w_ -1 and visual distance judgements. Error bars represent the SEM and the grey areas represent the 95% confidence interval.


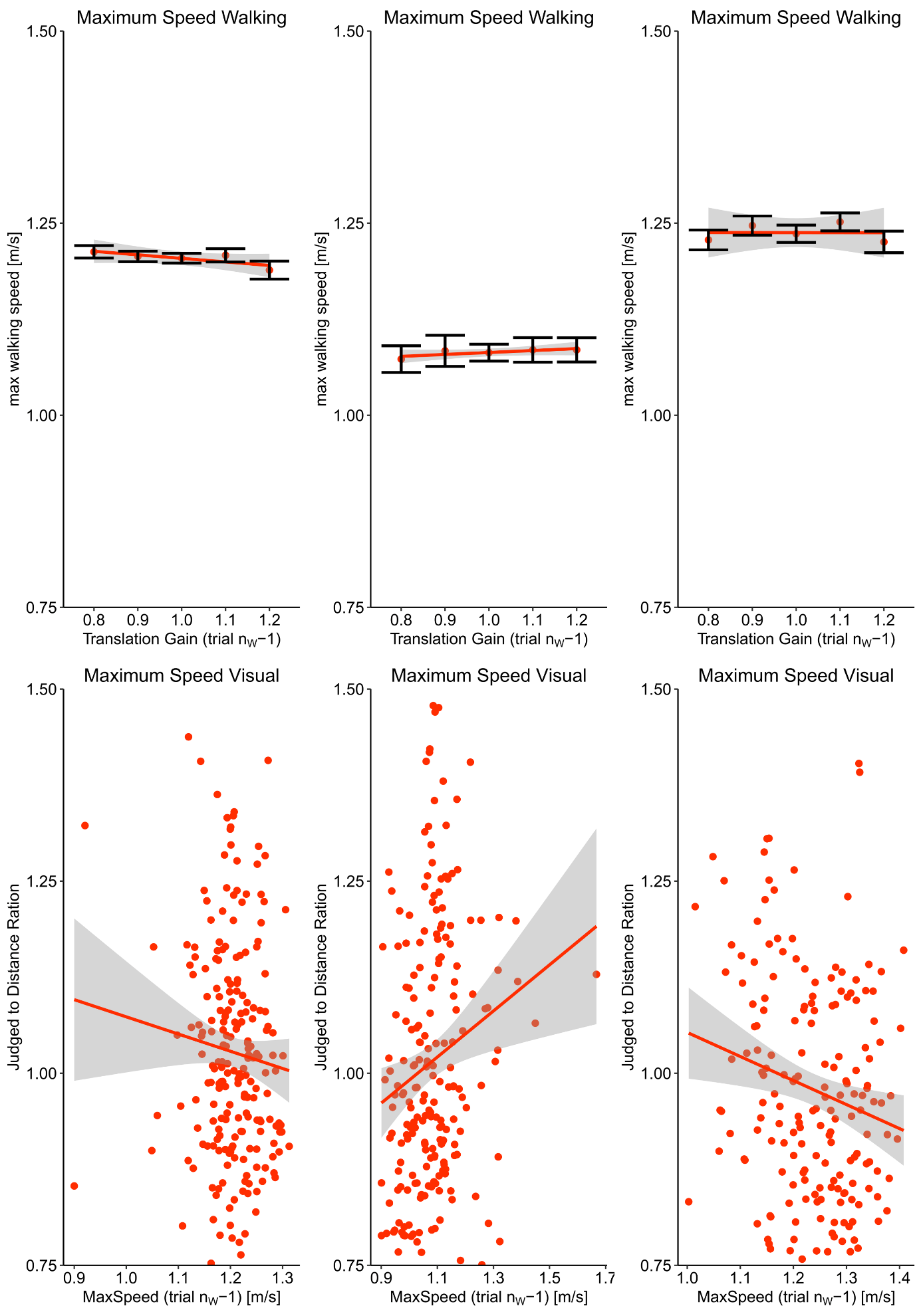


Figure S7 Maximum walking speed results of Experiment 3 for three example participants. Upper panel: Regression between the translation gain in trial n_W_ and maximum walking speed walking distance estimations in trial n_W_. Lower panel: Serial dependencies quantified by the regression between maximum walking speed trial n_w_ -1 and visual distance judgements. Error bars represent the SEM and the grey areas represent the 95% confidence interval.


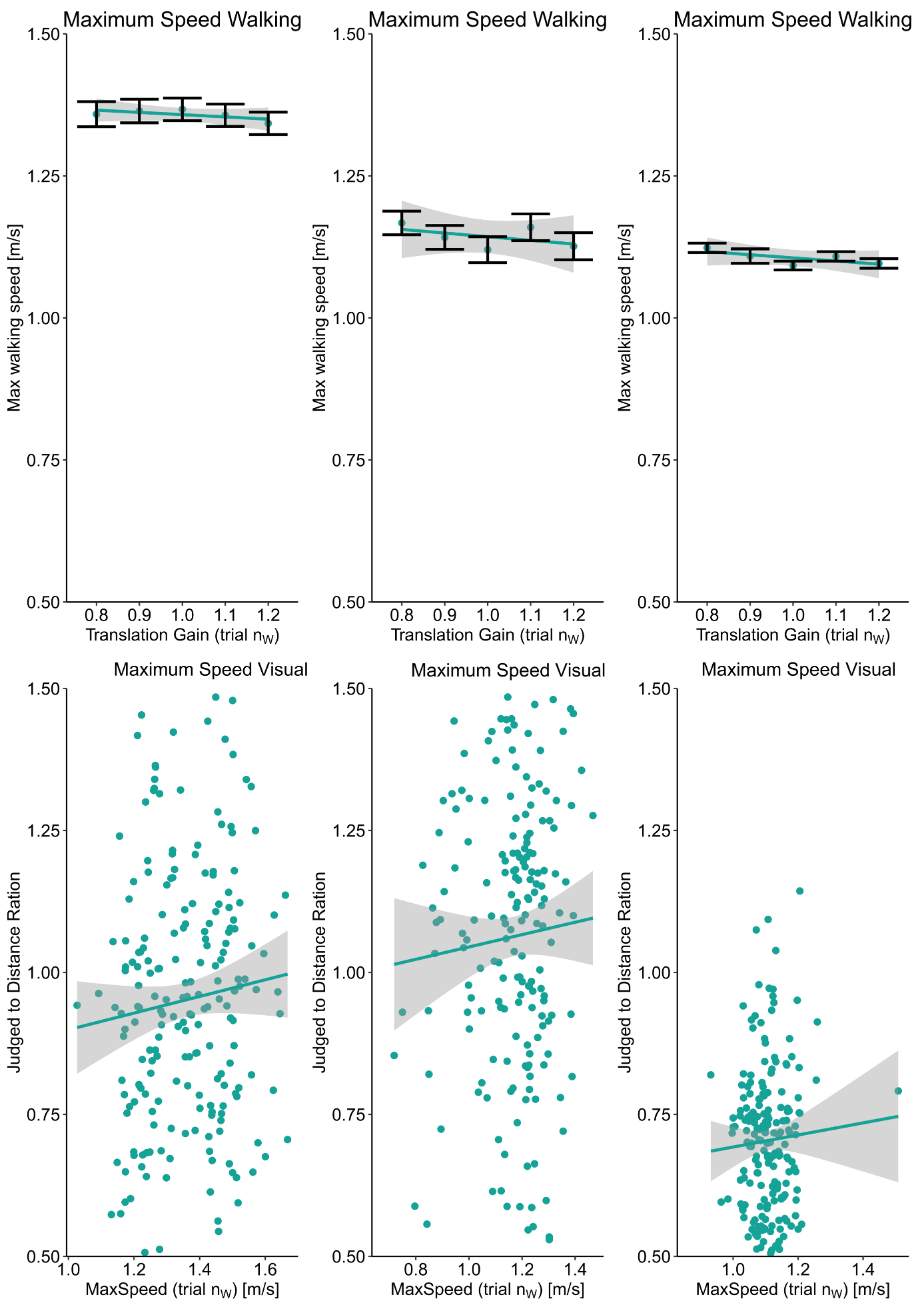


Figure S8 Maximum walking speed results of Experiment 4 for three example participants. Upper panel: Regression between the translation gain in trial n_W_ and maximum walking speed walking distance estimations in trial n_W_. Lower panel: Serial dependencies quantified by the regression between maximum walking speed trial n_w_ -1 and visual distance judgements. Error bars represent the SEM and the grey areas represent the 95% confidence interval.


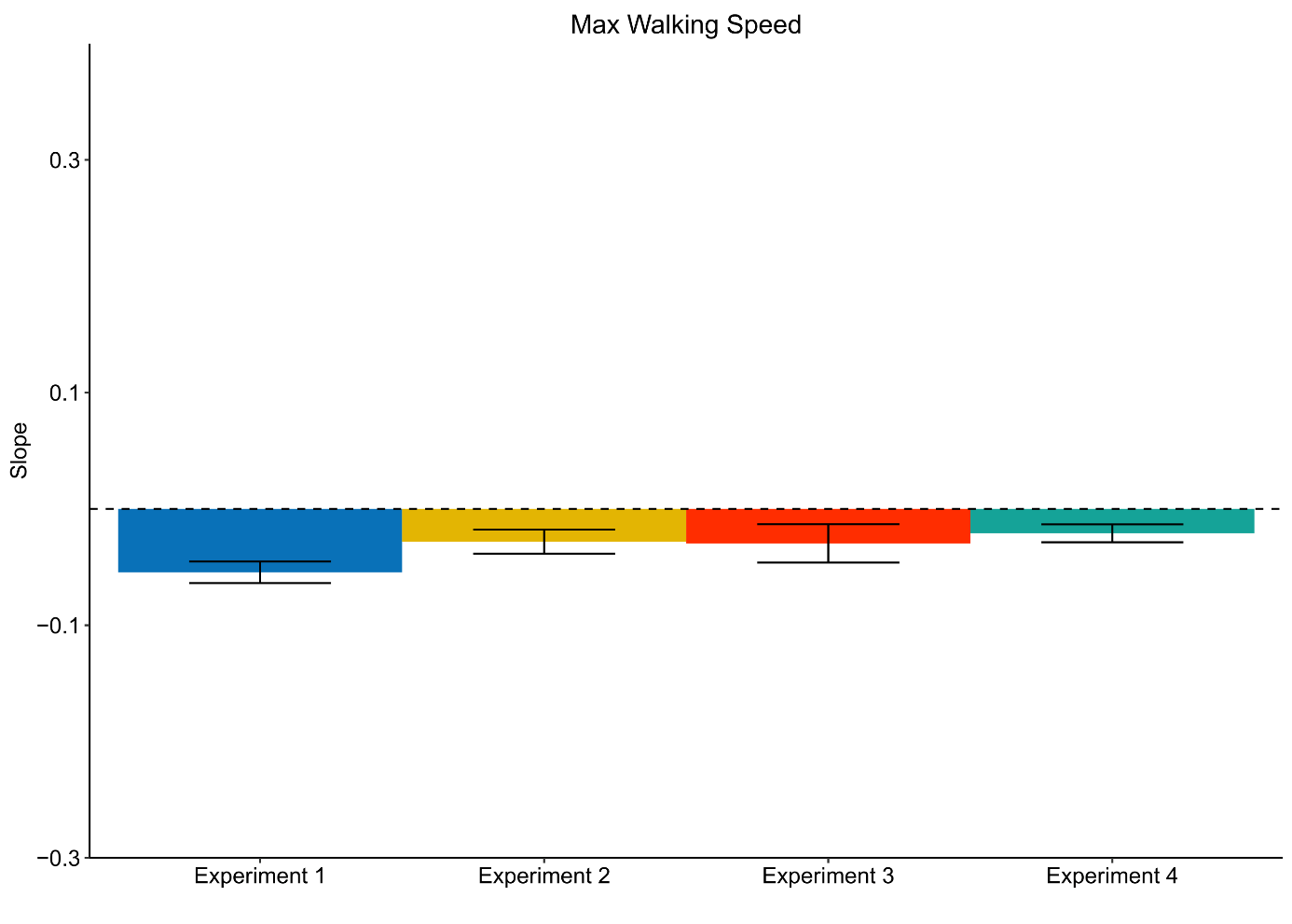


Figure S9. Average slopes representing the maximum walking speed during walking trials as a function of the translation gain in trial n_w_ for all experiments. Error bars represent the SEM.


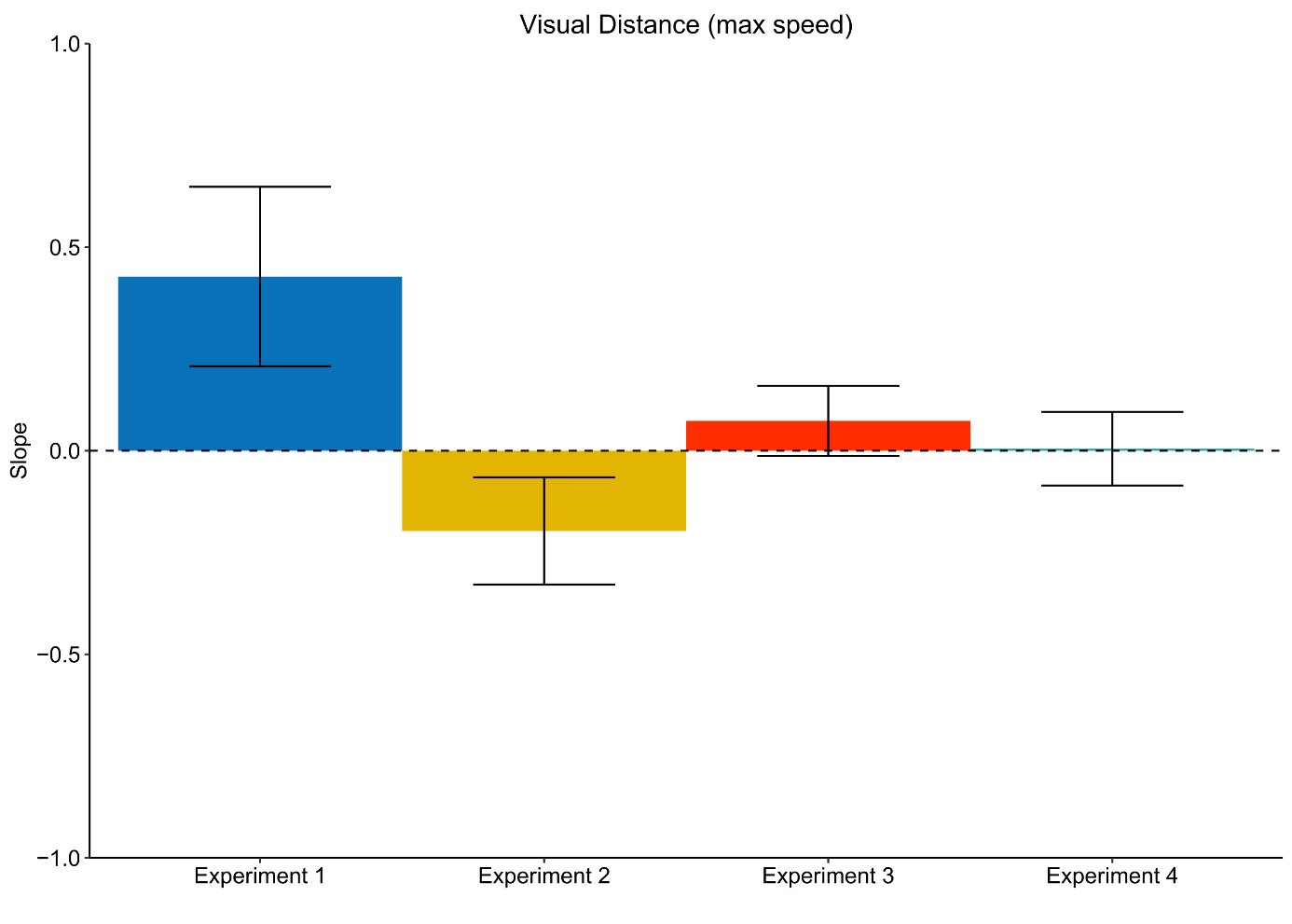


Figure S10. Average slopes representing the visual distance judgement as a function of the translation gain in trial n_w_-1 for all experiments. Error bars represent the SEM.


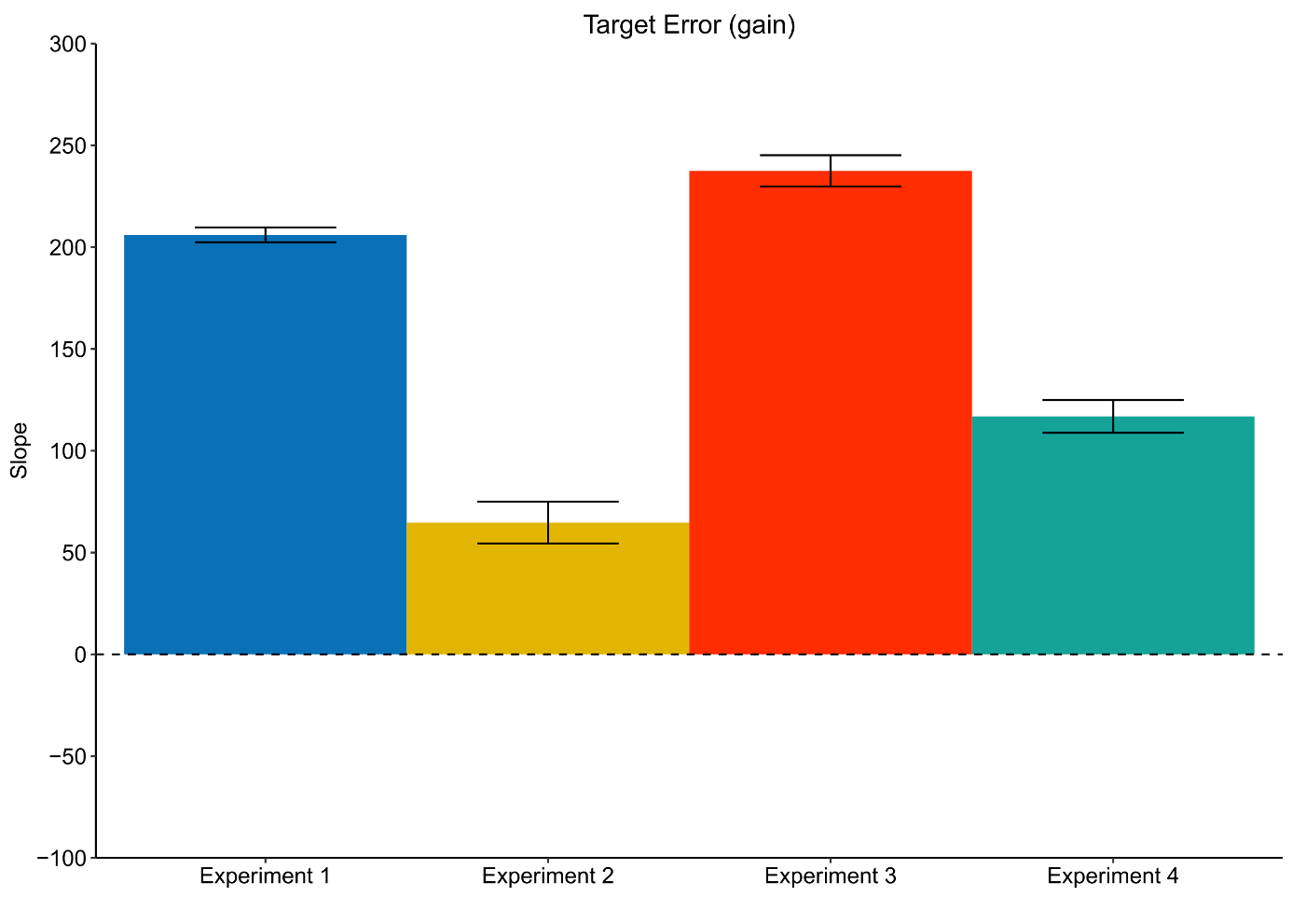


Figure S11. Average slopes representing the target error during walking trials as a function of the translation gain in trial n_w_ for all experiments. Error bars represent the SEM.


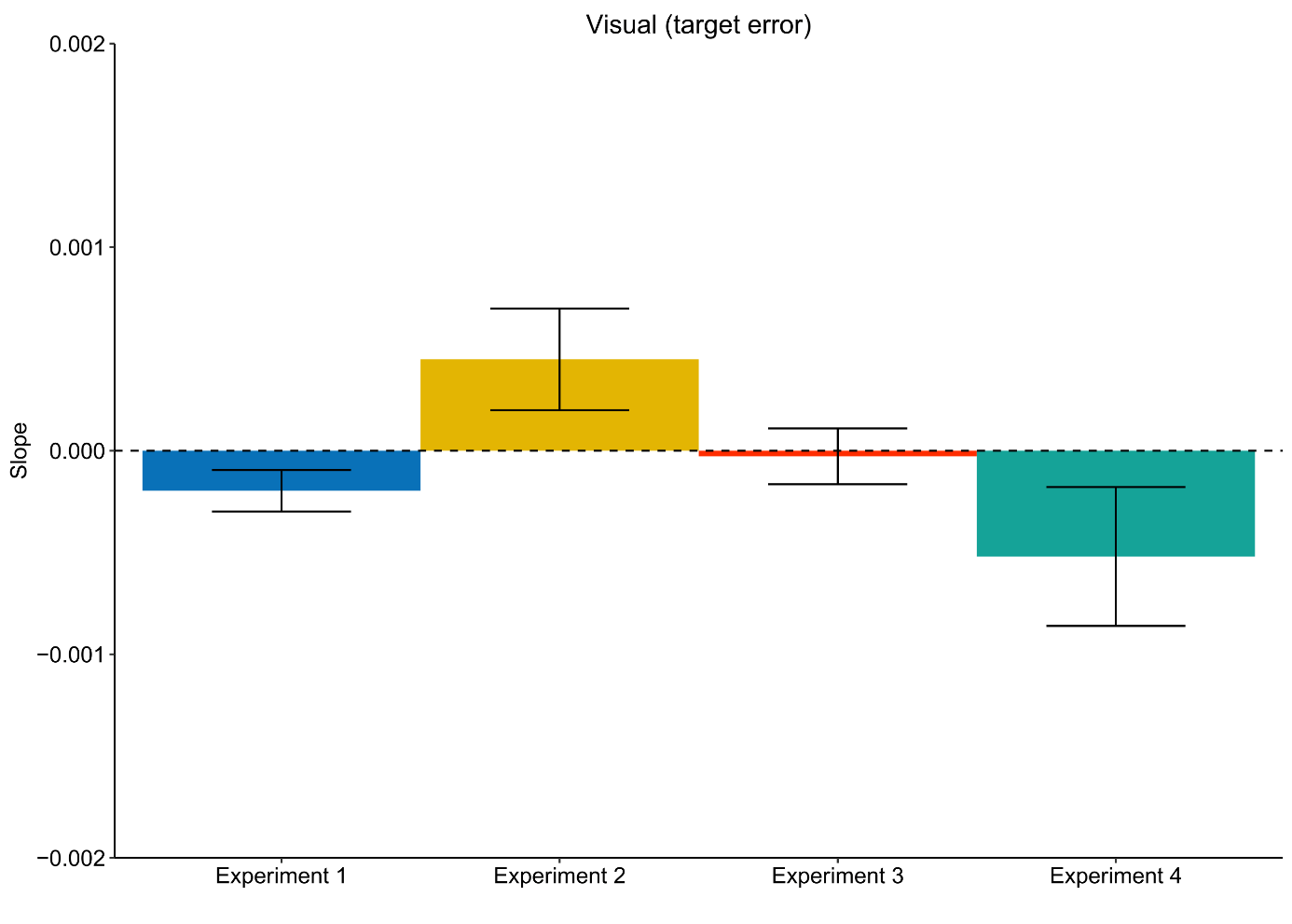


Figure S12. Average slopes representing the visual distance judgements trials as a function of the target error in trial n_w_-1 for all experiments. Error bars represent the SEM.
